# The Aβ40 and Aβ42 peptides self-assemble into separate homomolecular fibrils in binary mixtures but cross-react during primary nucleation†
†Electronic supplementary information (ESI) available. See DOI: 10.1039/c4sc02517b


**DOI:** 10.1039/c4sc02517b

**Published:** 2015-05-08

**Authors:** Risto Cukalevski, Xiaoting Yang, Georg Meisl, Ulrich Weininger, Katja Bernfur, Birgitta Frohm, Tuomas P. J. Knowles, Sara Linse

**Affiliations:** a Lund University , Biochemistry and Structural Biology , Chemical Centre , Lund , Sweden . Email: Sara.Linse@biochemistry.lu.se; b Cambridge University , Chemistry Department , Lensfield Road , Cambridge , UK; c Department of Biophysical Chemistry , Center for Molecular Protein Science , Lund University , Lund , Sweden

## Abstract

The assembly of proteins into amyloid fibrils, a phenomenon central to several currently incurable human diseases, is a process of high specificity that commonly tolerates only a low level of sequence mismatch in the component polypeptides. However, in many cases aggregation-prone polypeptides exist as mixtures with variations in sequence length or post-translational modifications; in particular amyloid β (Aβ) peptides of variable length coexist in the central nervous system and possess a propensity to aggregate in Alzheimer's disease and related dementias. Here we have probed the co-aggregation and cross-seeding behavior of the two principal forms of Aβ, Aβ40 and Aβ42 that differ by two hydrophobic residues at the C-terminus. We find, using isotope-labeling, mass spectrometry and electron microscopy that they separate preferentially into homomolecular pure Aβ42 and Aβ40 structures during fibril formation from mixed solutions of both peptides. Although mixed fibrils are not formed, the kinetics of amyloid formation of one peptide is affected by the presence of the other form. In particular monomeric Aβ42 accelerates strongly the aggregation of Aβ40 in a concentration-dependent manner. Whereas the aggregation of each peptide is catalyzed by low concentrations of preformed fibrils of the same peptide, we observe a comparably insignificant effect when Aβ42 fibrils are added to Aβ40 monomer or *vice versa*. Therefore we conclude that fibril-catalysed nucleus formation and elongation are highly sequence specific events but Aβ40 and Aβ42 interact during primary nucleation. These results provide a molecular level description of homomolecular and heteromolecular aggregation steps in mixtures of polypeptide sequence variants.

## Abstract 2

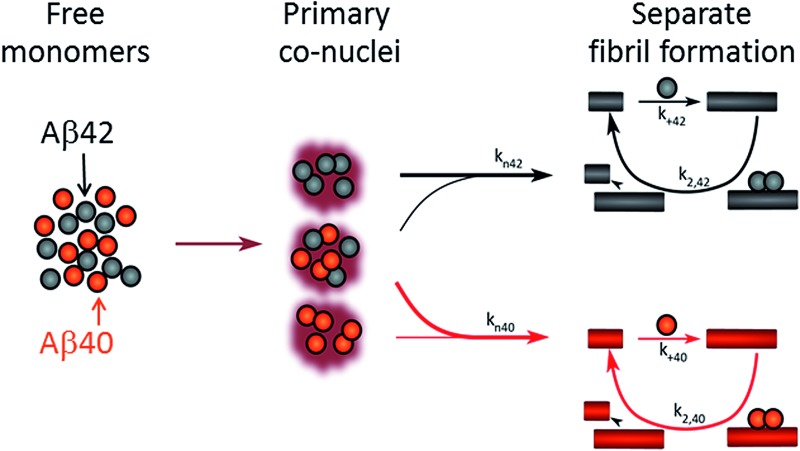
Reaction network starting from monomer mixtures of Aβ40 and Aβ42. Interaction at the level of primary nucleation only accelerates Aβ40 fibril formation. Separate fibrils form as secondary nucleation and elongation are highly specific.

## Introduction

Alzheimer's disease (AD) is the most common form of dementia and affects millions of people worldwide. The pathology behind this devastating disease includes self-assembly of the normally soluble amyloid β peptide (Aβ)^1–4^ into aberrant aggregates, in particular amyloid fibrils. Sporadic AD is the most common form of the disease and is thought to arise due to an imbalance between production and clearance of Aβ during aging.^5,6^ The Aβ peptide is generated by proteolysis from a larger transmembrane protein, the amyloid precursor protein (APP).^5^ In the amyloidogenic pathway APP is mainly cleaved before Asp1 of the Aβ-domain by β-secretase^5^ and the subsequent proteolysis with γ-secretase gives rise to peptides with a variety of C-terminal lengths.^7–9^ Aβ40 is the more common peptide, while the additional hydrophobic residues make Aβ42 more aggregation prone and it is more closely linked to the disease.^10^ *In vitro*, Aβ42 monomer is soluble up to *ca.* 0.1–0.2 μM (ref. 11) and at higher concentration it aggregates into well-ordered β-sheet-rich fibrillar structures. Amyloid plaques found in the brain of AD patients contain fibrillar Aβ. However, recent evidence suggests that smaller diffusible assemblies are likely to be the toxic species causing synaptic and neuronal loss.^10,12–15^ It has also been proposed that the aggregation process, rather than a specific aggregated form of the peptide, may be the critical and toxic event.^16,17^ The coexistence of several Aβ peptides differing in length by one or a few amino acids, and the connection between disease progression and both the total Aβ concentration and the Aβ42 fraction motivates studies of co-aggregation and cross-seeding behavior among those peptides. Co-aggregation refers to the formation of joint aggregates of any size and cross-seeding is the ability of aggregates of one peptide to promote the conversion of soluble peptides of the other type into growing aggregates. Due to the strong association of Aβ aggregation with neurodegeneration processes, it is important to understand at a molecular level the mechanism of aggregation in peptide mixtures. To this end it is crucial to determine whether there is discrimination or cooperation between peptides of different lengths for each microscopic step underlying the aggregation process.

Amyloid formation from peptides is a process of high specificity. A large number of human proteins are prone to self-assemble in the form of amyloid fibrils.^18,19^ These ordered fibrillar aggregates are tightly packed repetitive structures in which each peptide displays an identical segment that forms an interface for interaction with copies of itself and all peptides in the aggregate are in-registry.^20^ The extent of co-aggregation and cross-seeding between peptides and proteins with differing sequences has been studied in a number of cases.^21–27^ However, in general mixed fibrils of more than one protein or peptide are rarely observed.^27,28^ It is therefore of interest to define under which scenarios pure or mixed aggregates are formed in binary mixtures of peptides and proteins and to determine the level of mismatch tolerated for co-aggregation of Aβ variants. Of particular interest are aggregation processes in mixtures of the two major isoforms, *i.e.* Aβ40 and Aβ42. Two limiting scenarios can be envisaged, on the one hand formation of separate fibrils and no perturbation of the kinetics of aggregation, *i.e.* total inertness to the co-existence; and on the other hand formation of joint fibrils and perturbations of the kinetics. Intermediate scenarios between these limiting cases are possible, including the formation of separate fibrils in conjunction with perturbations of the kinetics, which would lead to kinetic effects observed as changes in the time-resolved aggregation data.

Aggregation in Aβ40:Aβ42 mixtures has previously been studied using thioflavin T fluorescence, electron paramagnetic resonance and nuclear magnetic resonance spectroscopy, as well as turbidity assays.^29–36^ Results from these studies indicated that the aggregation of Aβ42 is retarded by the presence of Aβ40, while Aβ42 may accelerate Aβ40 aggregation and that there is some degree of overall cross-seeding between the peptides.^24,29–34^ Some investigators have discussed that Aβ40 and Aβ42 might form mixed fibrils and these studies have revealed many surprising findings and explored the phenomenology associated with mixed aggregation.^30,32,35^ However, a common picture has not emerged regarding the relative effectiveness of cross-seeding and self-seeding, possibly due to the use of different peptide concentrations or co-solvents in different studies or the use of synthetic peptides, a factor that may introduce additional sequence heterogeneity and thus influence the kinetics or the equilibrium distribution.^37^ Some studies report that Aβ42 fibrils seed aggregation of Aβ40 (ref. 29 and 31–33) or that Aβ40 fibrils seed the aggregation of Aβ42.^29,32^ However, to our knowledge the underlying mechanism of aggregation in mixtures of Aβ42 and Aβ40 has not been elucidated.

**Fig. 1:**
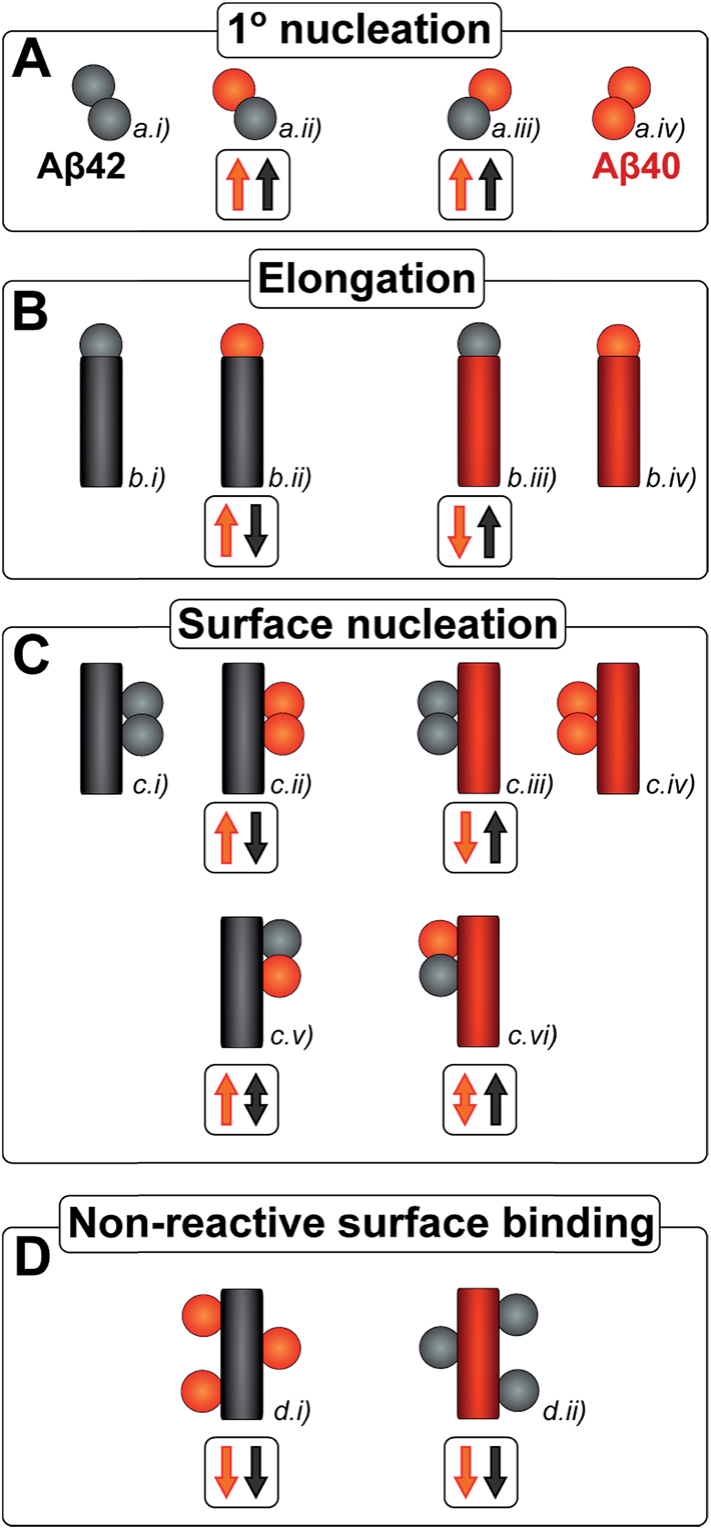
All simple (cross) reaction processes. A graphical depiction of the various simple reactions involving monomers or fibrils from either of the two protein species. These mechanisms combine to yield the overall reaction network of aggregation. For the processes which involve two different protein species, the expected effect on the aggregation propensity, compared to the aggregation of each protein on its own, is given by the arrows below the mechanism. For example an upwards red arrow denotes that the process in question is expected to increase the aggregation propensity of the red protein in the presence of black protein. A double arrow signifies that an effect in either direction is possible, depending on the specific conditions.

The kinetic analysis of mixtures requires simultaneous preparation of both peptides as highly pure monomers. Moreover, to reach a mechanistic understanding of the aggregation process in a binary peptide mixture, an essential ingredient is a prior knowledge of the aggregation mechanism of each peptide taken in isolation. For Aβ42 as well as Aβ40, the overall growth curves have a sigmoidal shape including a lag phase, a growth phase and a plateau when the reaction comes to completion at late times. Detailed analysis of large sets of kinetic data show that the same composite steps underlie the aggregation mechanism for both peptides.^38,39^ In particular, for each peptide the process is governed by a double nucleation mechanism;^40^ primary nucleation of monomers in solution is slow (Fig. 1A.i & iv), while secondary nucleation on the surface of already formed aggregates is a more rapid process (Fig. 1C.i & iv). Primary nucleation refers to nucleation reactions involving monomeric peptide only, whereas secondary nucleation generates new aggregates in a process involving both monomers and fibrils of the same peptide. Thus, fibrils provide a catalytic surface for nucleation from monomers. As fibrils are formed at an early stage in the process, the surface-catalyzed secondary nucleation soon becomes the dominant route to generate new aggregates.^38,39,41^ Global kinetic analysis has revealed that the molecular level origin of the overall slower aggregation of Aβ40 stems from the lower rate of both primary and secondary nucleation events relative to the situation found for Aβ42, with primary nucleation being most compromised.^39^ Fragmentation is another type of secondary process, but under quiescent conditions, such as in the present and previous mechanistic studies, it was found to be a negligibly slow process.^38,39,41^

Kinetic studies have further revealed that the microscopic processes of primary and secondary nucleation and growth take place during all three phases of the characteristic sigmoidal aggregation.^42^ Thus, for instance, the characteristic lag-time prior to the observation of significant quantities of aggregates by bulk assays, is not solely dependent on primary nucleation rates as a simple sequential picture of the aggregation reaction might suggest, but rather is affected also by fibril elongation and secondary nucleation.^38,39,41^ Different microscopic processes, however, govern the overall behavior at each stage, as determined by the rate constants and concentrations of reacting species. Since all these microscopic processes are in principle amenable to perturbation by another peptide in the same solution (Fig. 1), the quest for a molecular level description of co-aggregation represents a complex task. As a strategy towards addressing this challenge, we have applied a set of experiments to isolate the specific contributions to primary and secondary nucleation by varying either the concentration of pre-formed aggregates of a given type or by varying the concentration of monomeric precursor peptide in the initial reaction mixtures, and have then followed the aggregation kinetics as a function of these different initial conditions. The use of integrated rate laws, which have recently become available for the study of amyloid formation, then allows us to connect the observed kinetic behavior on the bulk scale to the microscopic events that govern the aggregation reaction. This approach represents the conventional workflow of mechanistic analysis in small molecule chemistry, but has to date been challenging to apply to aggregating protein systems due to the difficulty of obtaining highly reproducible kinetic data and the lack of suitable rate laws required by such an analysis.

The current work provides a detailed mechanistic study of aggregation processes in binary mixtures of Aβ40 and Aβ42. In order to obtain data of suitable quality, all peptides and peptide mixtures were prepared in highly pure form in a phosphate buffer without co-solvents, and recombinant peptides were used to ensure high level of sequence homogeneity. The kinetics of aggregation were monitored using a thioflavin T (ThT) fluorescence assay;^11^ we have optimized the assay conditions (see methods) to obtain highly reproducible data and have verified that the ThT fluorescence is proportional to the concentration of aggregates and thus a faithful reporter of the progress of the reaction. The secondary structure was studied as a function of time for peptide mixtures using circular dichroism (CD) spectroscopy. In order to gain insights into the morphology of the aggregates formed in pure and mixed samples, we used *cryo* transmission electron microscopy (*cryo*-TEM) that does not require specific staining. The monomer composition was studied at several time points using isotope labeling, mass spectrometry and nuclear magnetic resonance (NMR) spectroscopy. Using this strategy, we are able to analyze whether there is cooperation in each of the molecular level processes underlying the overall aggregation mechanism, and to evaluate the role of a molecular mismatch in C-terminal length. Our results show that Aβ40 and Aβ42 interact significantly only at the level of primary nucleation, leading to a two-stage aggregation process and preferential formation of separate fibrils.

## Results

**Fig. 2:**
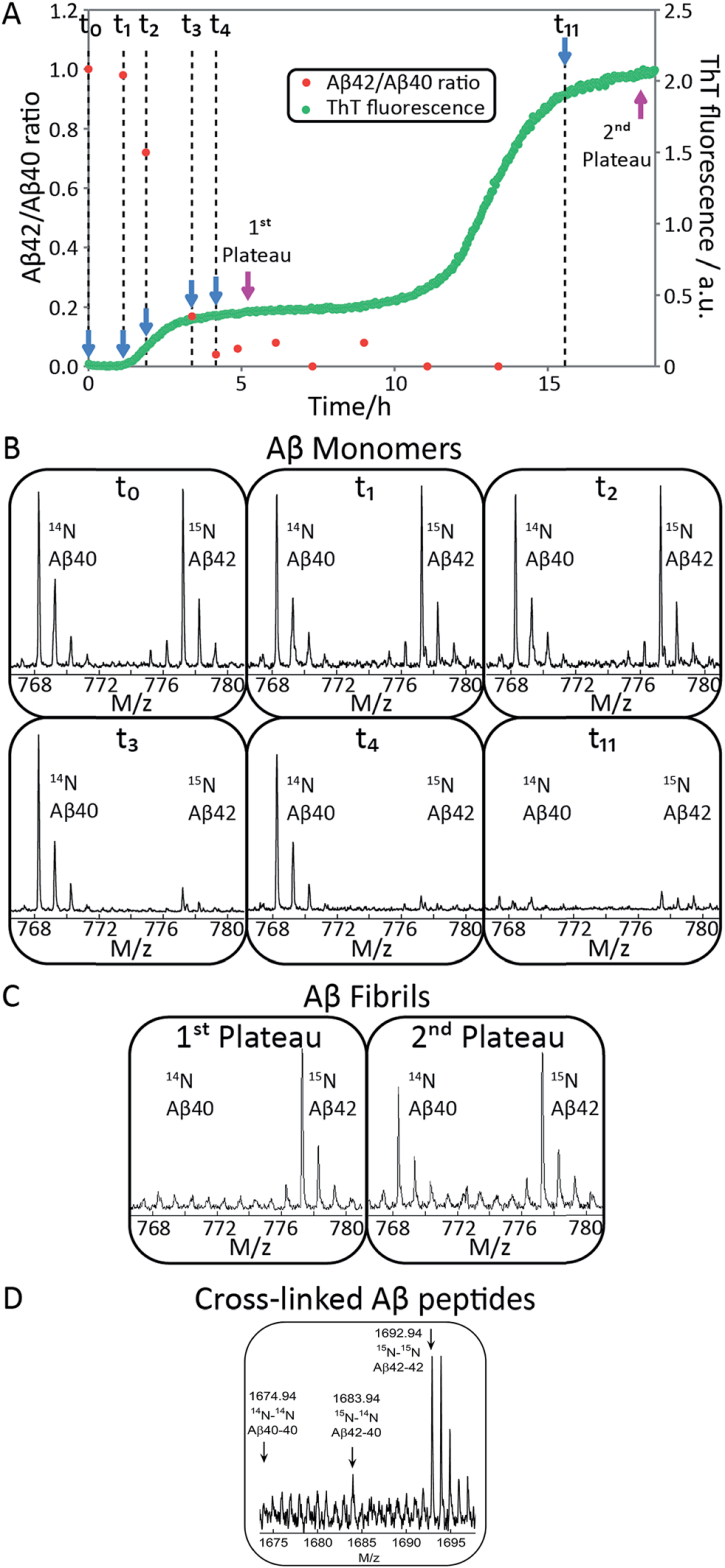
Aβ42 and Aβ40 monomer depletion during separate transitions. (A) The aggregation of a mixture of 1.5 μM Aβ42 and 1.5 μM Aβ40 monomer was monitored by ThT fluorescence (green), and the ratio of Aβ42/Aβ40 monomer concentration remaining in solution at 11 time points measured by mass spectrometry (red). The process displays two transitions by ThT fluorescence. Aβ42 monomer is depleted during the first transition and Aβ40 monomer is depleted during the second transition. (B) The corresponding mass spectra from six of the time points are shown below the aggregation curve. The Aβ42/Aβ40 ratio is close to 1 at *t*_0_ and at *t*_1_ (end of lag-phase) and decreases to close to 0 after the first plateau has been reached. Aβ42 monomer is depleted during the first sigmoidal transition and Aβ40 monomer is consumed during the second sigmoidal transition, suggesting the formation of separate fibrils. (C) Mass spectra of fibrils collected at the first and second plateau. Aβ42 fibrils are the main components at the first plateau while both Aβ40 and Aβ42 fibrils are present at the second plateau. This indicates that the first transition is mainly due to the aggregation of Aβ42 while Aβ40 aggregation mainly is responsible for the second transition. (D) Mass spectra of cross-linked Aβ peptides. Samples were cross-linked at different time points during the lag phase and digested by trypsin. A sum over seven repeats is shown. Cross-linked Aβ42–42 is clearly observed (1692.94 corresponds to two M1-5 fragments with a single crosslink) and there is a weak signal corresponding to cross-linked Aβ40–42 (1683.94).

**Fig. 3:**
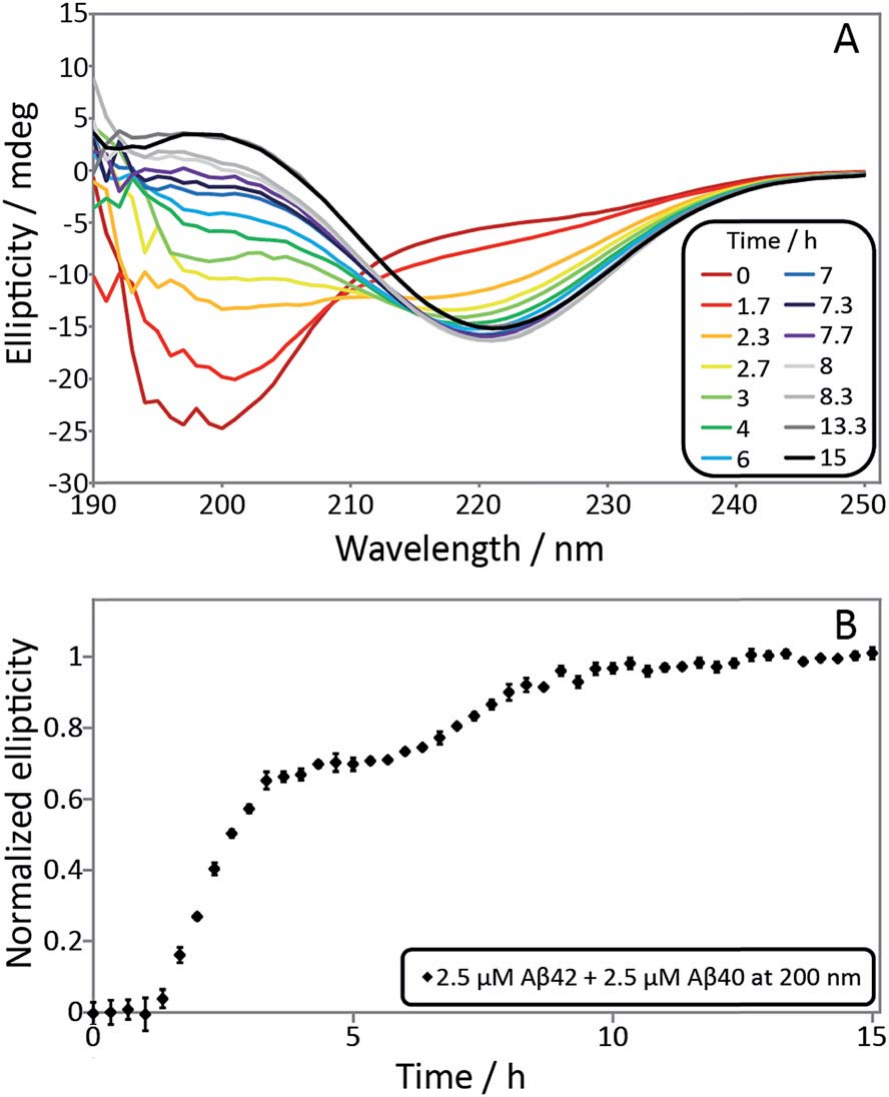
(A) Secondary structural change monitored by far-UV CD spectroscopy for a mixture of 2.5 μM Aβ42 and 2.5 μM Aβ40. Far-UV CD spectra as a function of time monitors the transition from random coil to β-sheet. The minimum shifts from around 200 nm at time zero to 218 nm after around 4 h and is then shifted to 220 nm after 15 h. (B) Normalized ellipticity at 200 nm as a function of time. Error bars represent the standard deviation. The sample contains 5 mM sodium phosphate and 40 mM NaF, pH 7.4.

**Fig. 4:**
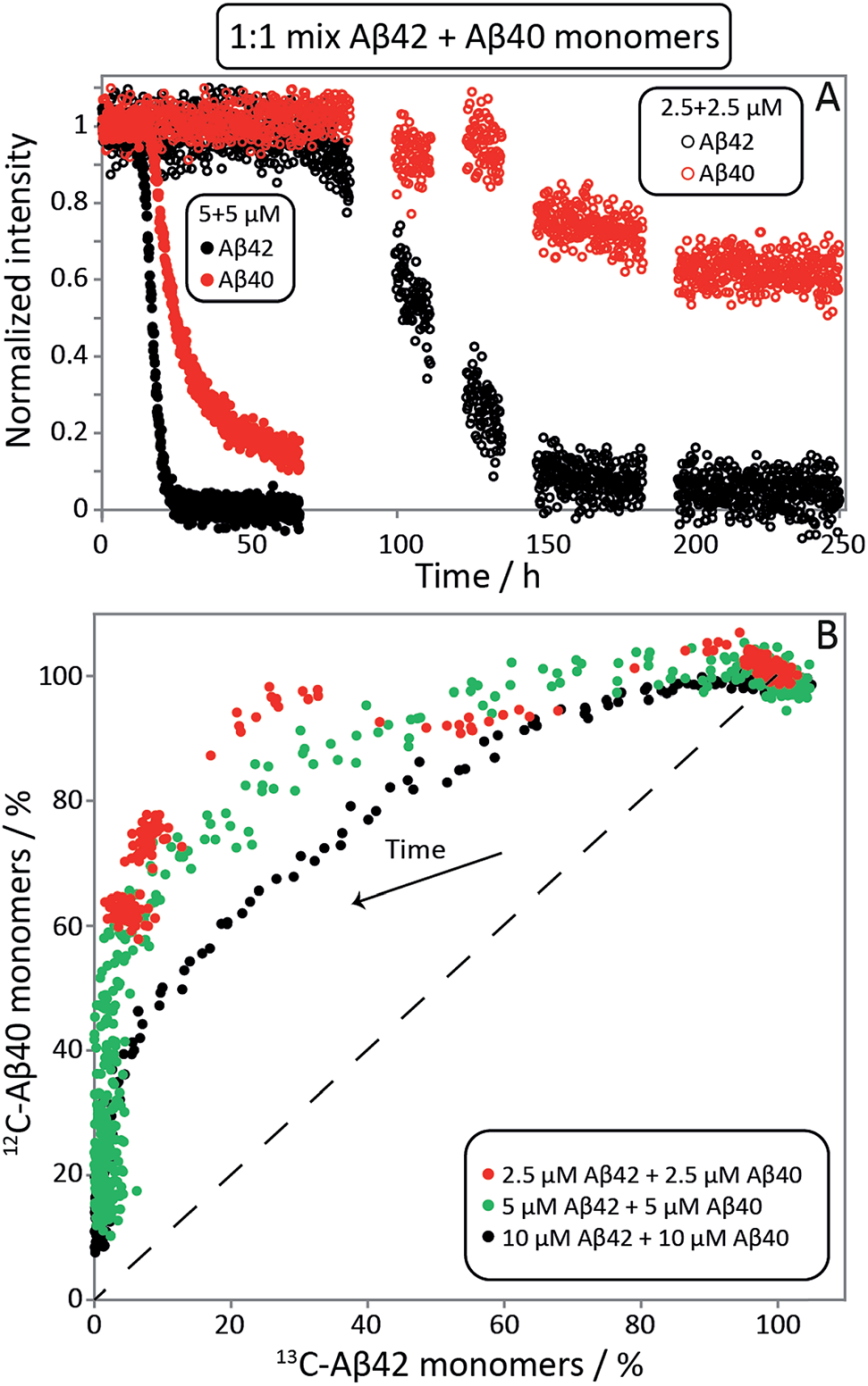
Aβ42 and Aβ40 monomer concentrations monitored by NMR spectroscopy. (A) ^13^C-Aβ42 and ^12^C-Aβ40 monomer concentrations as a function of time, derived from the intensities of ^13^C- or ^12^C-filtered methyl-protons signals, respectively, in experiments starting from 1 : 1 monomer mixtures. In the lag phase, both monomers are close to 100% of the initial value. Thereafter the consumption of Aβ42 is faster than Aβ40. (B) Aβ40 monomer concentration as a function of Aβ42 monomer concentration, expressed as % of the concentration at time zero. Depletion increases over time, shown as an arrow. The dashed line illustrates the hypothetical scenario of equal consumption rate of both peptides over the entire time course. For the 2.5 μM + 2.5 μM sample every data point shown is an average over 11 time points. The samples contain 20 mM sodium phosphate, 200 μM EDTA, 0.02% NaN_3_, pH 7.4.

**Fig. 5:**
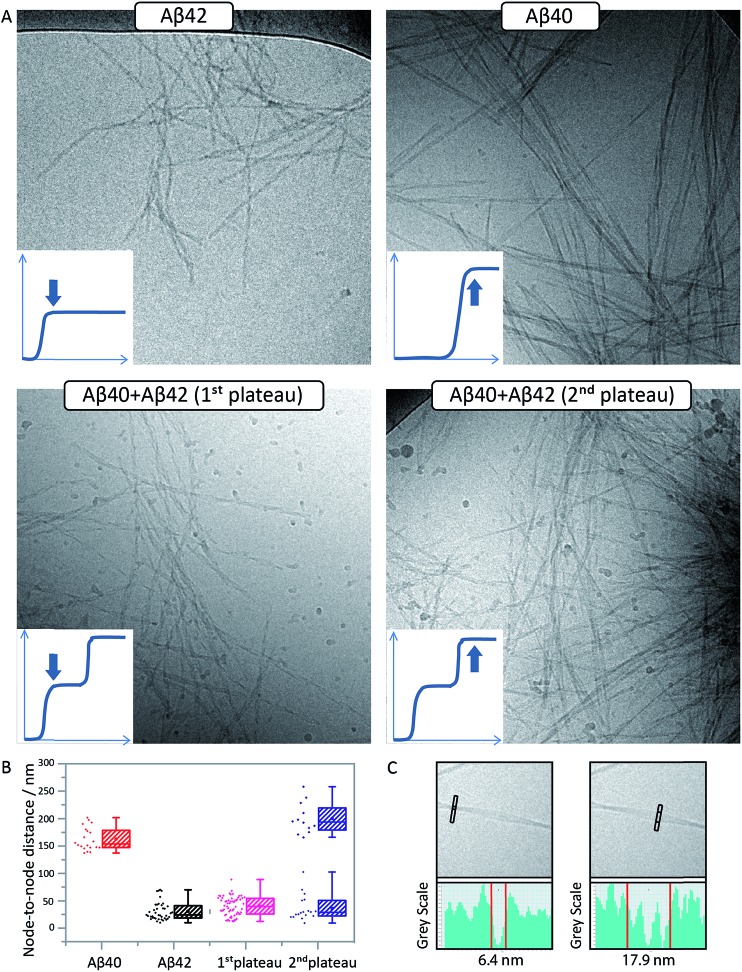
Aβ42 and Aβ40 form distinct, separate fibrils. (A) *Cryo*-TEM images of pure Aβ42 fibrils (top left), pure Aβ40 fibrils (top right) and fibrils formed in an equimolar mixture of Aβ42 and Aβ40 at the 1^st^ (lower left) and 2^nd^ (lower right) plateaus are shown. Pure Aβ42 fibrils are short and twisted with a shorter helical repeat. Pure Aβ40 fibrils are larger, straighter and thicker. The samples contain 5 μM ThT, 20 mM sodium phosphate, 200 μM EDTA, 0.02% NaN_3_, pH 7.4. (B) An analysis of the node-to-node distance yields a quantitative measure for the two morphologies confirming that fibrils collected at the 1^st^ plateau have similar morphology as Aβ42 fibrils, while the fibrils collected at the 2^nd^ plateau display both types of morphologies. Points show individual measurements. The box contains the middle 50%, the line indicates the median, and the whiskers include all data points. (C) Zoom-ins of pure Aβ40 fibrils illustrating the method used for finding the nodes for node-to-node distance measurements. The nodes were defined as points along the fibril where a single minimum gray scale was observed (left), while between the nodes several minima are observed which is due to multiple protofilaments involved in the same fibril (right).

**Fig. 6:**
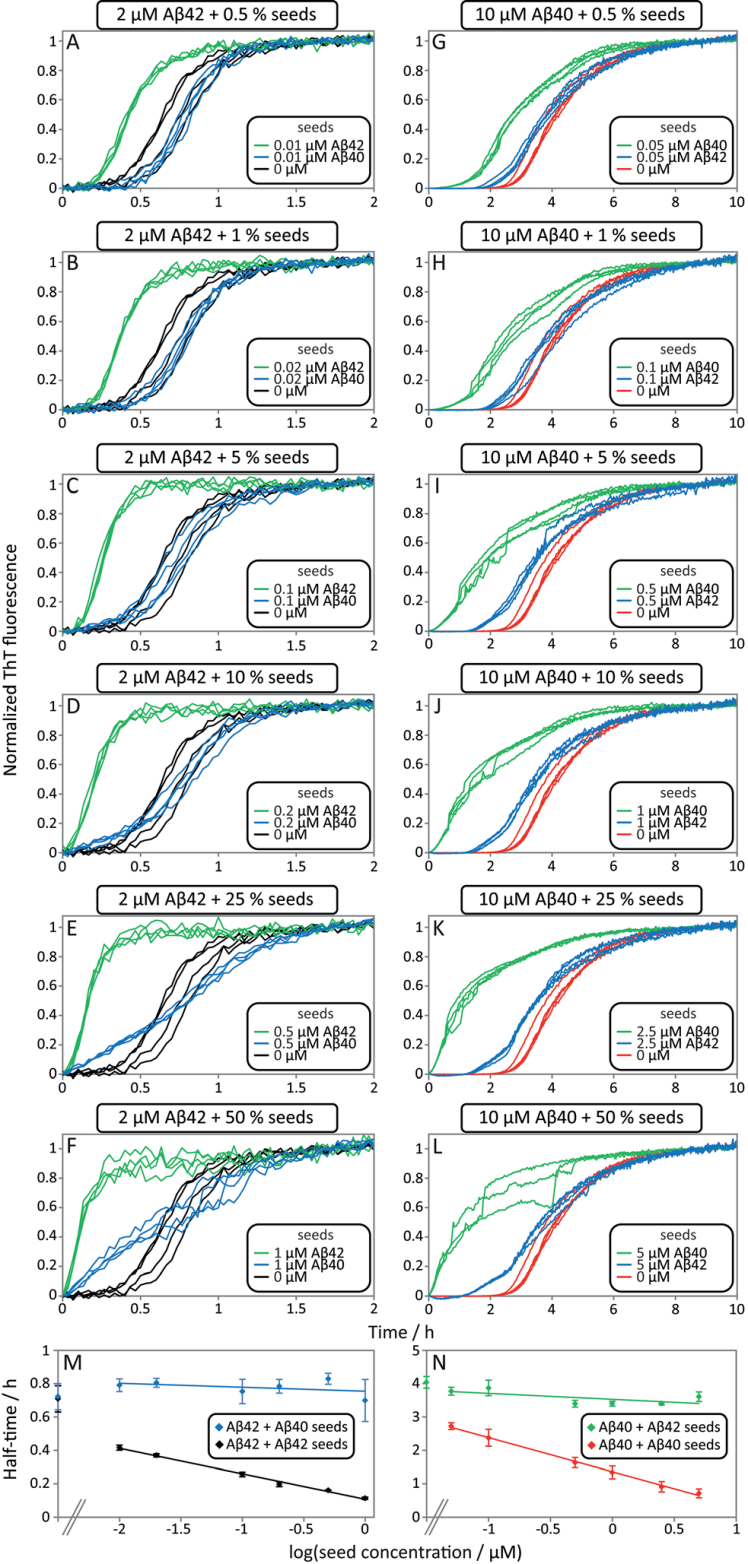
Self and cross-seeding experiments of Aβ42 and Aβ40. (A–F) and (G–L) Self and cross-seeding of Aβ42 and Aβ40, respectively, in 20 mM sodium phosphate, 200 μM EDTA, 0.02% NaN_3_, pH 7.4 with 5 μM ThT. Four repeats of each condition are shown. With increasing self seed concentration the lag-phase decreases until the sigmoidal shape disappears. The self-seeding is much more efficient than the cross-seeding. At high seed concentrations (10–50%) the ThT fluorescence increases earlier with cross seed compared to no seed, but without affecting the half-time. (M and N) Half-time as a function of the logarithm of the seed concentration. The self-seeding data displays clear concentration dependence while the cross-seeding data lack this property. Each data point is an average of at least three replicates with error bars representing the standard deviation.

**Fig. 7:**
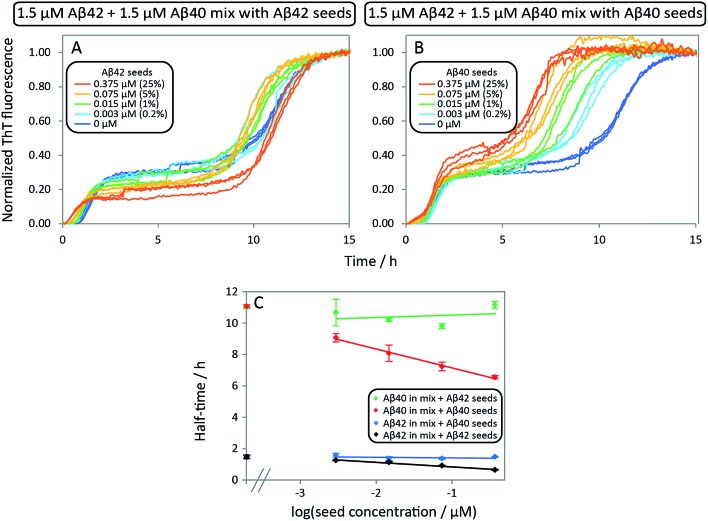
Aβ42 and Aβ40 mixture seeded by preformed Aβ42 seeds and Aβ40 seeds, respectively. (A) 1.5 μM Aβ42 + 1.5 μM Aβ40 mixture with Aβ42 seeds. The first transition of the mixture is only catalyzed by adding Aβ42 seeds while there is no effect on the second transition (corresponding to Aβ40 aggregation). (B) 1.5 μM Aβ42 + 1.5 μM Aβ40 mixture with Aβ40 seeds. The second transition (corresponding to Aβ40 aggregation) is only catalyzed by adding Aβ40 seeds while there is no effect on the first transition (corresponding to Aβ42 aggregation). The catalytic effect is more pronounced with increasing seed concentration both in A and B. (C) Half-time as a function of the logarithm of the seed concentration. The extent of the decrease of the half-time is seed concentration dependent and is in agreement with the self-seeding of Aβ42 and Aβ40 (Fig. 6). Each data point is an average of three replicates with error bars representing the standard deviation. The samples contain 5 μM ThT, 20 mM sodium phosphate, 200 μM EDTA, 0.02% NaN_3_, pH 7.4.

**Fig. 8:**
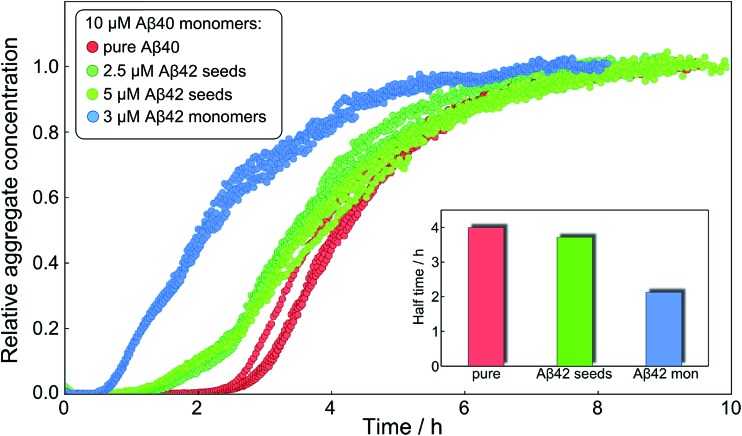
Only monomeric Aβ42 affects Aβ40 aggregation strongly. The normalized aggregation curves of 10 μM monomeric Aβ40 alone (red), with 2.5 μM Aβ42 seeds (dark green), 5 μM Aβ42 seeds (light green) and with 3 μM Aβ42 monomer (blue). The inset shows the approximate half times under the three different conditions. Whilst the aggregation of Aβ40 is affected only slightly by 2.5 μM or 5 μM fibrillar Aβ42, it is affected strongly at similar concentration, 3 μM, of monomeric Aβ42.

**Fig. 9:**
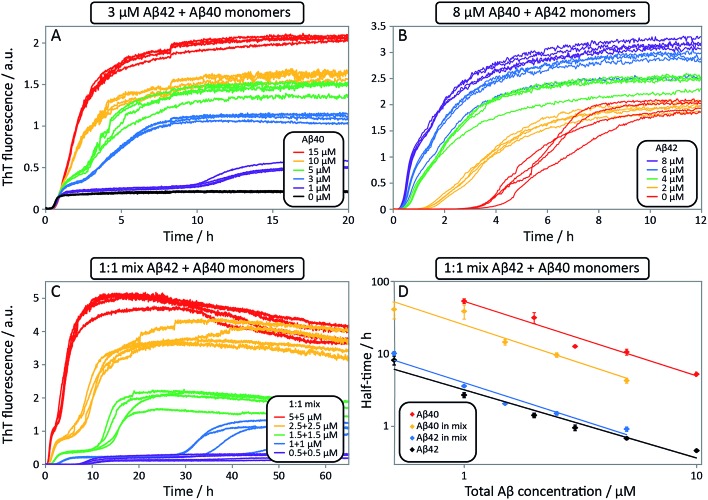
Aggregation kinetics for mixtures of Aβ42 and Aβ40, and of pure peptides. ThT fluorescence intensity as a function of time. (A) 3 μM Aβ42 and varied Aβ40 concentrations. The effect of Aβ40 on the aggregation of Aβ42 is small (see first sigmoid). (B) 8 μM Aβ40 and varied Aβ42 concentrations. Aβ40 is accelerated significantly by Aβ42, seen by a shorter lag-phase of the second transition as Aβ42 concentration is increased. Four replicates of each concentration are shown. (C) ThT fluorescence intensity as a function of time for equimolar mixtures at five different concentrations. Four replicates of each concentration are shown. (D) The half-time for the kinetics in C as a function of total Aβ concentration. The half-time is defined as the point in time where the ThT fluorescence is half-way between baseline and first plateau values for Aβ42 or first and second plateau values for Aβ40. Each data point is an average of four replicates with error bars representing the standard deviation. The solid lines are power functions fitted to the experimental data. Aβ40 aggregation in the mixture is accelerated by Aβ42 seen by a shorter half-time (compare red with yellow) while Aβ42 aggregation is not affected significantly by Aβ40 (compare black with blue). All samples contain 5 μM ThT, 20 mM sodium phosphate, 200 μM EDTA, 0.02% NaN_3_, pH 7.4.

**Fig. 10:**
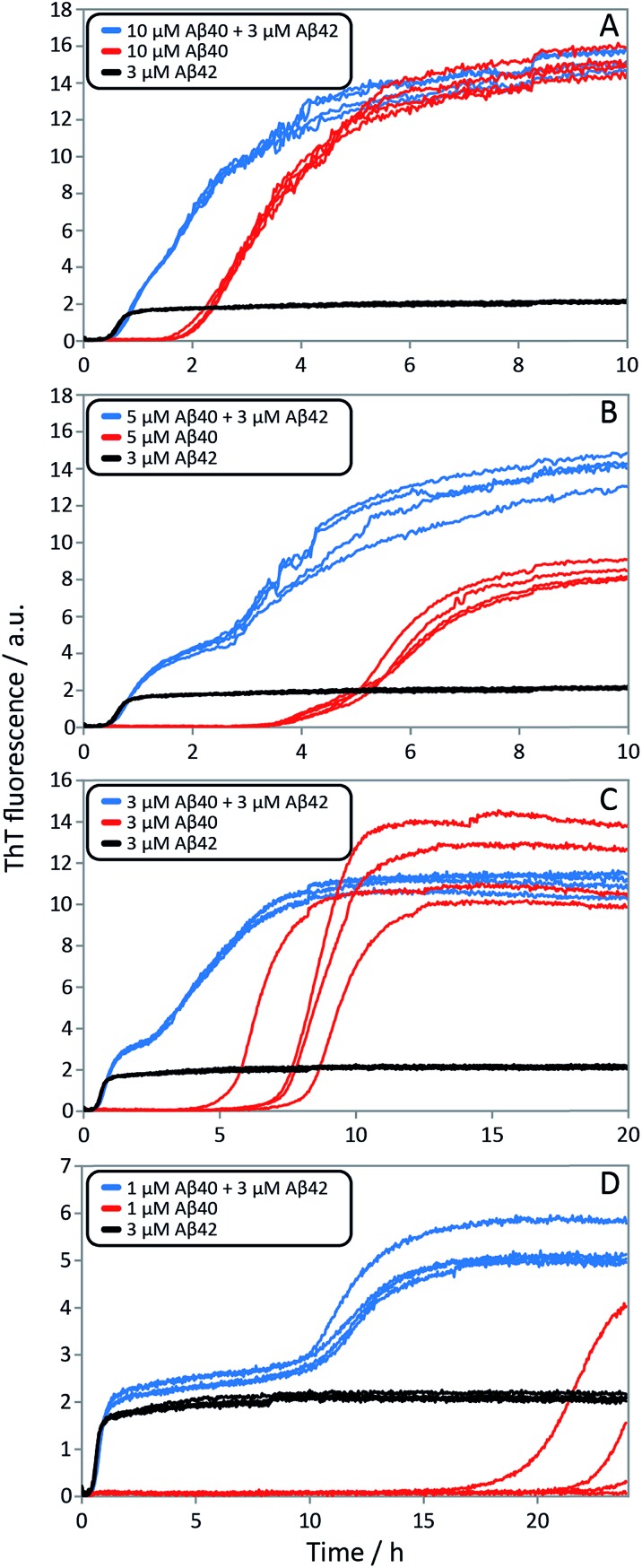
Aggregation kinetics for mixtures of Aβ42 and Aβ40, and of pure peptides. ThT fluorescence intensity is recorded as a function of time for mixtures at four different Aβ42 : Aβ40 ratios (blue), 3 μM Aβ42 (black) and Aβ40 at the same concentration as in the respective mixture (red). The accelerating effect on Aβ40 is significant for all ratios and especially at lower ratios the two-stage aggregation process becomes more evident (compare blue with red). Aβ40 has little effect on 3 μM Aβ42 (compare blue with black). Four replicates of each condition are shown. The samples contain 5 μM ThT, 20 mM sodium phosphate, 200 μM EDTA, 0.02% NaN_3_, pH 7.4.

**Fig. 11:**
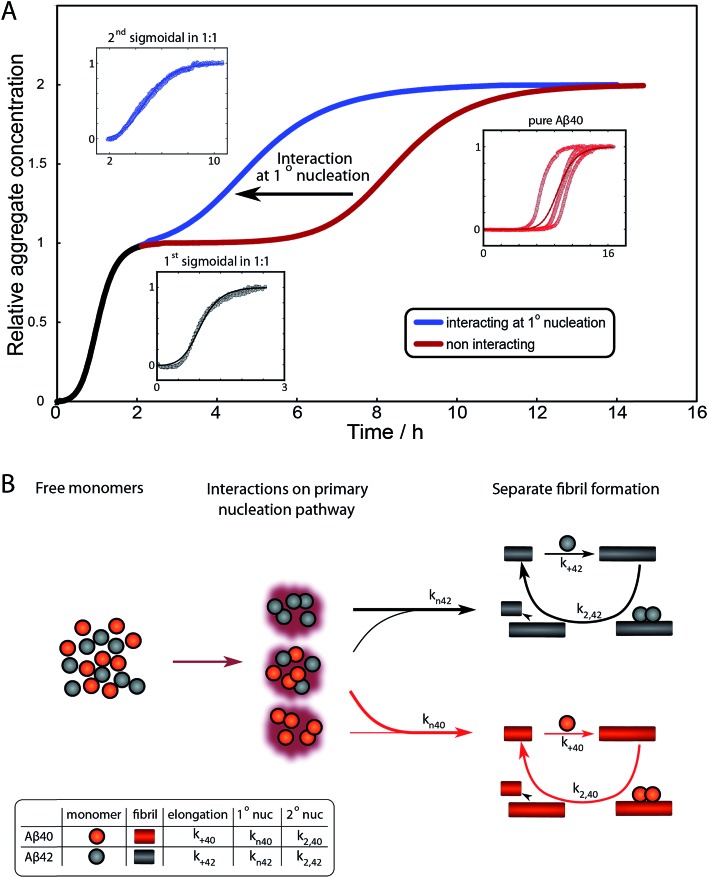
Simulated curves and reaction network. (A) Simulated aggregation curves starting from mixed monomers of Aβ42 and Aβ40 at 3 μM, showing the effect of an interaction at primary nucleation only. The simulated curve was obtained by combining the sigmoidals obtained from the fits for the aggregation of Aβ42 (black) with that for the aggregation of either pure Aβ40 (red) or Aβ40 in a mixture (blue), where the fits to the two Aβ40 datasets vary only in their primary nucleation constant. Hence the black and red line shows the expected behaviour if the two peptides were to aggregate completely independently, the black and blue curve shows the expected behaviour if Aβ42 monomers promote the primary nucleation of Aβ40 monomers, increasing the nucleation rate by approximately 2 orders of magnitude. The insets show the fits to the corresponding data. (B) The reaction network showing the aggregation of the two peptides, incorporating the dominant process of interaction at the level of primary nucleation. This cooperative nucleation pathway may contribute nuclei to the formation of both types of fibrils, however, the effect is only significant for Aβ40, as this has a very low primary nucleation rate in its pure form.

The co-aggregation and cross-seeding behavior of Aβ42 and Aβ40 were studied using a ThT fluorescence assay,^11^ CD and NMR spectroscopy, mass spectrometry as well as *cryo*-TEM. ThT undergoes a red-shift in its emission spectrum with an enhanced quantum yield,^43,44^ when it non-covalently binds to amyloid fibrils. This property has widely been used to monitor amyloid formation.^45^ ThT assays commonly suffer from a lack of reproducibility, which we have minimized in our work by optimizing the purity of the reactants and removing the presence of contaminants such as small quantities of pre-formed oligomers or micro air bubbles, and by optimizing the ThT concentration.^38,39^ The mechanism of aggregation for pure Aβ40 and Aβ42 has previously been established at pH 7.4 and 8.0, respectively.^38,39^ Therefore Aβ42:Aβ40 mixtures were investigated at both these pH values. Data at pH 7.4 are shown in Fig. 2–11, and data at pH 8.0 are show in ESI Fig. S3–S5.†

### Double transitions in the aggregation kinetics of Aβ40:Aβ42 mixtures

In order to determine whether or not mixed fibrils form in the presence of both peptides, we first probed the aggregation time course for reactions initiated with 1 : 1 mixtures of freshly isolated monomers of Aβ40 and Aβ42 (Fig. 2A, 9C, S1A and S2A†). The ThT fluorescence intensity displays two distinct transitions as a function of time for these samples. This feature is observed for all concentrations explored in this study, but the intermediate plateau is more pronounced for the lower peptide concentrations. The data further show that aggregation reactions initiated from mixtures of Aβ42 and Aβ40 follow characteristic time courses of a strikingly different shape relative to those obtained from the individual pure peptide samples; the latter are characterized by a single sigmoidal transition, while the former display two distinct transitions. The finding of two sequential transitions for mixtures of Aβ40 and Aβ42 is an indication that two distinct aggregation processes are taking place on different time scales within these samples. At pH 8.0 the intermediate plateau is even more pronounced relative to that observed at pH 7.4. Our objective is to characterize these well-defined changes in terms of microscopic processes underlying mixed aggregation phenomena.

In order to confirm the biphasic nature of the aggregation process, we employed CD spectroscopy (Fig. 3 and S3†) to follow the progressive conversion of the peptides from their soluble states, consisting predominantly of random coil structure, to the fibrillar form which is β-sheet rich, as reported previously.^46^ The CD spectra in Fig. 3 show that the system consists of a mixture of unstructured peptides and β-sheet rich fibrils at the first plateau. At the second plateau the soluble peptides have converted to β-sheet rich conformations, in agreement with the ThT fluorescence data. We note that that the comparison between the times observed in the ThT assay and the CD assay is complicated by the differences in the surface chemistry of the containers used in both assays, UV-transparent quartz cuvette for the CD measurements and PEG-coated multi-well plates for the ThT measurement. Moreover, the CD measurements are conducted with stirring present to prevent sedimentation. The emergence of a double sigmoidal in both cases is a strong indication that this is a feature characteristic of aggregation from mixtures of Aβ40 and Aβ42.

### Isotope labeling identifies the first transition as Aβ42 and the second as Aβ40

To identify unambiguously the peptide aggregating at each of the two transitions observed both by CD spectroscopy and ThT fluorescence, we used selective labeling of the peptides with stable isotopes for mass-spectrometric identification (Fig. 2). To this effect, the ThT experiment was repeated while initiating the aggregation reaction from equimolar monomer mixtures of ^15^N-Aβ42 and ^14^N-Aβ40. The data obtained for experiments starting with 1.5 μM of each peptide are shown in Fig. 2A and those for 2.5 or 5 μM of each peptide in Fig. S1 and S2.† During the time course of the reaction, we removed aliquots which were then subjected to centrifugation to sediment any fibrillar material. The ratio of the concentrations of Aβ42 and Aβ40 monomers remaining in solution was determined from the supernatant by mass spectrometry after digestion by trypsin, which cleaves after arginine and lysine residues. This approach measures the intensity of the ratio of ^15^N-Aβ(M1-5) and ^14^N-Aβ(M1-5) peaks, originating from Aβ42 and Aβ40, respectively. In this manner, we circumvent the uncertainties inherent in the quantification of peptides by mass spectrometry due to the difference in ionization of full-length Aβ42 and Aβ40. Examples of mass spectra of several time points are shown in Fig. 2B, and the resulting Aβ42/Aβ40 ratio is shown as a function of time in Fig. 2A. The results for the 1.5 + 1.5 μM sample (Fig. 2A and B) show that at the beginning of the experiment and during the lag phase, the Aβ42/Aβ40 ratio is close to 1.0, but that this ratio decreases as the ThT fluorescence increases. When the first ThT plateau is reached, the Aβ42/Aβ40 ratio has dropped to approximately zero indicating that the Aβ42 monomers are depleted during the first transition to form Aβ42 fibrils. This finding also holds for the 2.5 + 2.5 and 5 + 5 μM samples (Fig. S1 and S2†).

Samples withdrawn at the first and second plateau were also filtrated through 0.2 μm spin filter. The trapped fibrils were washed by adding Milli-Q water, whereby any species smaller than 0.2 μm were washed away and fibrils retained. These fibrils were digested by trypsin and the identity of the N-termini of peptides in the fibrils was determined by mass spectrometry (Fig. 2C). The results shows that fibrils collected at the first plateau are mainly composed of Aβ42 monomers. From the small peaks and level of noise at the position where the Aβ40 signal should appear, we can deduce that less than 13% of Aβ40 is contained in the fibrils at this stage. Both Aβ42 and Aβ40 are detected in the fibril sample collected at the second plateau. These findings are consistent with the monomer depletion measurements and indicate that the first transition in the co-aggregation kinetics is mainly due to the Aβ42 aggregation while Aβ40 aggregation is responsible for the second transition.

Samples withdrawn at eight different time points during the initial lag phase were cross-linked, followed by tryptic digestion and analysis by mass spectrometry. Although nuclei are transient species of low abundance, signals from Aβ42–42 nuclei (at *m*/*z* = 1692.94, corresponding to cross-linked ^15^N-Aβ(M1-5)–^15^N-Aβ(M1-5)) were clearly detected in most repeats but signals from Aβ42–40 co-nuclei (at *m*/*z* = 1683.94, corresponding to cross-linked ^15^N-Aβ(M1-5)–^14^N-Aβ(M1-5)) are weak or absent in most repeats in line with their even lower abundance and transient nature. Signal from Aβ40–40 nuclei is not seen in any repeat. In Fig. 2D we show the sum over seven repeats that show weak signal at the *m*/*z* value expected for Aβ42–40 co-nuclei, in addition to the relatively strong signal from cross-linked Aβ42–42 dimer.

When studied by ThT fluorescence, the amplitude of the first transition is lower relative to that of the second transition. This finding is in agreement with the lower quantum yield for ThT bound to Aβ42 compared to Aβ40 fibrils, which is consequently seen as a difference in the amplitude between the signal originating from the aggregation of pure Aβ42 and pure Aβ40 when studied at the same concentration. The opposite holds for the CD data, however, in this case the lower amplitude for the second transition most likely originates from signal loss due to light scattering being more significant the more fibrils are present in solution. These results demonstrate that the first transition corresponds to the formation of Aβ42 fibrils and the second to the formation of Aβ40 fibrils. Thus, Aβ42 and Aβ40 preferentially form separate fibrils, at least to within the accuracy of our mass spectrometry measurements.

### Aβ42 *vs.* Aβ40 monomer depletion – NMR spectroscopy

NMR spectroscopy offers the possibility to study the aggregation of Aβ42 and Aβ40 separately in mixed samples using isotope labels. The Aβ42 and Aβ40 monomer concentrations in 1 : 1 mixtures of ^13^C-Aβ42 and ^12^C-Aβ40 were monitored as a function of time by alternating application of pulse sequences that selectively detect signals from ^13^C-bound (Aβ42) or ^12^C-bound (Aβ40) protons. Three experiments were carried out starting from monomer mixtures of 2.5, 5 or 10 μM of each peptide. While lower concentrations offer better separation between the two processes as found by ThT fluorescence (Fig. 2, S1 and S2†), the two higher concentrations were included because of the relatively poor sensitivity of NMR spectroscopy. The presence of quartz surface, a different geometry, a high surface area to volume ratio, and a smaller air–water-interface to volume ratio, will have a significant effect on the sensitive aggregation reaction and may explain the altered and much slower kinetics in NMR tubes compared to non-binding PEG-coated plates. As these factors may affect the individual underlying microscopic processes differently, only a qualitative comparison with the experiments in non-binding plates is warranted. The use of a drastically different sample environment thus allows us to test whether the separate aggregation processes for Aβ42 and Aβ40 as observed in the experiments monitored by ThT, mass spectrometry and CD spectroscopy are artifacts of the situations during those experiments. In Fig. 4A, the Aβ42 and Aβ40 monomer concentrations are shown as a function of time as extracted from the NMR signal intensities. In Fig. 4B the Aβ40 monomer concentration is plotted as a function of Aβ42 monomer concentration. In all cases, we observe a lag-phase during which the monomer concentration stays close to 100% of the initial value for both peptides, and the data show very clearly that the depletion of Aβ42 monomer starts several hours earlier than the Aβ40 monomer depletion, in agreement with the mass spectrometry data (Fig. 2). The differences in consumption rates between Aβ40 and Aβ42, and the dependence of the Aβ40 consumption rate on Aβ42 monomer concentration are also evident from Fig. 4B, in which the dashed line represents equal consumption rate of both peptides, a behaviour which would be expected for complete co-aggregation. The data deviate more from the equal consumption line, the lower the initial monomer concentrations, and the fraction of Aβ40 monomers left in solution when all Aβ42 monomers are consumed is higher the lower the starting concentration in the mixture. Thus, the aggregation of Aβ40 is accelerated by the presence of Aβ42 monomers, but not Aβ42 fibrils, implying cooperation during primary nucleation.

### Cryo-TEM analysis of fibril morphology confirms the formation of distinct fibrils

To confirm the formation of fibrils and to evaluate their morphology, samples were collected for *cryo*-TEM at the times corresponding to the first and second plateau in the ThT assay at pH 7.4 for the Aβ42:Aβ40 mixture and at the single plateau for pure Aβ42 and Aβ40 (Fig. 5A). The samples were prepared in the same way as for the kinetic studies and the total peptide concentration in all samples was 10 μM. As seen in Fig. 5A, there is a clear difference in morphology between Aβ40 and Aβ42 fibrils. The use of *cryo*-TEM allows us to circumvent the need for heavy metal staining, and any morphological differences observed are thus not related to differential stain uptake as can be the case for conventional negatively stained TEM. The Aβ40 fibrils appear larger, straighter and thicker than Aβ42, while the latter are more twisted with a shorter helical repeat clearly visible in the micrographs. The measurement of the typical helical half pitch (node-to-node distance) was performed using grey scale profiles (Fig. 5C) and was found to be 162 ± 21 nm for Aβ40 and 31 ± 17 nm for Aβ42, Fig. 5B. In the Aβ42:Aβ40 mixture there is a difference in the morphology of the fibrils depending on the stage at which the sample is collected. Samples collected at the first plateau display fibrils similar to the ones observed in the pure Aβ42 sample, and the node-to-node distance is also similar to Aβ42, 39 ± 17 nm, while at the second plateau there is a co-existence of the Aβ42-type fibrils with fibrils similar to the ones obtained in the pure Aβ40 sample, and the node-to-node distances fall into two distinct groups at 36 ± 23 nm and 199 ± 28 nm, Fig. 5B. *Cryo*-TEM therefore provides additional support for the finding that Aβ40 and Aβ42 undergo distinct self-assembly processes on separate time scales, even when present in binary mixtures.

### General principles of mixed aggregation kinetics

Amyloid fibrils, as linear aggregates, are able to grow from their ends by recruiting soluble peptides (Fig. 1B.i/iv). New fibrils can be formed by primary nucleation from soluble peptides (Fig. 1A.i/iv), a process which is commonly observed to be very slow, but can be catalyzed by the surfaces of existing fibrils in the form of secondary nucleation (Fig. 1C.i/iv). These processes have to date mainly been considered in systems which contain only a single aggregation prone polypeptide sequence. A full microscopic description of co-aggregation for systems which contain peptides with two or more distinct sequences is highly complex since it would have to account for all possible types of mixed aggregates and the rate constants that lead to their formation and growth. For the Aβ peptides studied in this work, we observe, however, that only homomolecular fibrils are formed. Thus in our analysis we considered one peptide species to act as a perturbation on the equations describing the other peptide in its pure form. To account for the changes upon introduction of another peptide, we consider separately the interactions of monomers amongst each other and of monomers with fibrils of either type. Thus, the processes which involve two different peptide species will affect the overall kinetics of aggregation relative to the aggregation of a single species of peptide. Depending on the specific manner of this interaction, the overall aggregation rate can be observed to either increase or decrease upon introduction of the second peptide species, as denoted by arrows in Fig. 1.

We first consider an interaction of peptides with differing sequences at the level of monomers. This process could promote the formation of nuclei (Fig. 1A.ii/iii), thereby speeding up the aggregation, or result in off-pathway mixed oligomers, thereby slowing the aggregation. The latter scenario is unlikely to be significant, as only a very small fraction of the peptides is found in oligomeric form at any given time during the aggregation reaction and off-pathway species would need to be present in sufficient quantities to deplete free monomer in order to slow the aggregation reaction.^38,39^ We note that although this process is represented in Fig. 1 by the interactions of two monomers, the mathematical model (see methods) also allows for interactions in bigger oligomers.

In our kinetic scheme we next consider the interactions of soluble peptides with fibril ends (Fig. 1B). Such interactions can lead to the growth of filaments through elongation. Since only homomolecular Aβ fibrils are observed in our experiments, we neglect the addition of a peptide with the incorrect sequence, but allow for the possibility that the presence of such a peptide could affect the rate at which the correct peptide can participate in fibril elongation.

Interactions with the surfaces of the fibrils can result in secondary nucleation (Fig. 1C.i/iv), the autocatalytic process that is responsible for the characteristic curve shapes, observed in the case where the monomer and fibril are of the same species. If monomer and fibril differ in sequence, several processes are possible: monomers can simply bind to the surface in a non-reactive manner (Fig. 1D), thereby depleting the monomer concentration of one species and the number of free surface sites for the other species, which will result in a decrease in aggregation rate for both proteins. Fibrils could also catalyze the formation of nuclei of the other species (Fig. 1C.ii/iii), thereby increasing the rate of aggregation for this peptide and potentially decreasing their own aggregation rate by blocking reactive surface sites for secondary nucleation. Lastly fibrils could catalyze the formation of mixed nuclei such as discussed above (Fig. 1C.v/vi). This effect could lead to both an increase or a decrease of the aggregation rate, depending on the competition between formation of these mixed nuclei and formation of pure nuclei and which type of fibril these mixed nuclei then go on to form.

These classes of molecular level events underlie a general description of mixed aggregation as summarised in Fig. 1. By controlling carefully the presence of either monomers or fibrils of both species, we will probe separately the contribution from these distinct processes and thereby build up a mechanistic picture of how the presence of Aβ40 monomers or fibrils perturbs the aggregation of Aβ42 and *vice versa*.

### Aβ42:Aβ40 cross-seeding *versus* self-seeding

In order to tackle the mechanistic complexity and detect which molecular level processes are affected by co-aggregation, we performed seeding and cross-seeding experiments. In these experiments, a well-defined quantity of pre-formed fibril seeds is introduced into the solution of the monomeric peptide to initiate the aggregation reaction. The approach allows the primary nucleation step to be circumvented since there are already fibrils present in the system at the beginning of the reaction. In the case of self-seeding it has been shown that at low seed concentrations, the growth of the seeds themselves is not sufficient to contribute significantly to the overall reaction rate; however, these seeds provide a surface which can act as a catalyst for the formation of new fibrils when secondary nucleation mechanisms are active. At high concentrations, by contrast, a sufficient quantity of fibrils is present that the consumption of monomer simply through the growth of the seed fibrils becomes the dominant contribution to the overall reaction. Thus, under low seed concentration conditions information can be obtained about the secondary nucleation rate, whereas at high seed concentrations, the rate is sensitive to the elongation rate. In the present experiments, we have extended this approach to evaluating the possible existence of cross-seeding, where the seed fibrils are not formed from the same peptide as the monomeric peptide in solution. To this effect, we studied the aggregation kinetics for samples with one peptide as freshly isolated monomer and the other peptide supplied as preformed seed fibrils.

This approach thus allows us to separately investigate the effect of fibrils of one species on the aggregation of the other species from monomer, significantly simplifying the interpretation of the data as the increase in the fluorescence signal is under these conditions only due to the aggregation of the protein present in monomeric form. Moreover, the other peptide is present only in its fibrillar form, allowing us to neglect monomer–monomer interactions between different peptide species. Once we have elucidated the cross-interactions between the monomeric peptides and fibrillar forms under these carefully controlled conditions, we will be in a position to analyse the full aggregation pathway from monomeric mixtures.

Data from self and cross-seeding experiments at pH 7.4 are shown in Fig. 6 and the data at pH 8.0 are found in Fig. S4.† Both Aβ42 (Fig. 6A–F) and Aβ40 (Fig. 6G–L) possess a high propensity towards self-seeding already at low seed concentration (0.5–10%) due to the presence of a surface-catalyzed secondary nucleation process that leads to an increased rate of creation of fibrils and consequently a shortened lag phase. In both cases of self-seeding, the lag phase is eliminated at high seed concentrations (10–50%) and the half-time is substantially reduced compared to the unseeded case (the half-time is the time at which half the peptide is in its aggregated form and can be used as a quantifier of a peptides aggregation propensity). In striking contrast, the cross-seeding data reveal very little effect due to the presence of the other peptide, as reflected in the unchanged half-times. In particular, in the low seed range the data for Aβ42 aggregation in the presence of 0.5–10% Aβ40 seeds closely superimpose with the data for non-seeded samples (Fig. 6A–D). This clearly shows that Aβ40 seeds present much less catalytic surface for nucleation of Aβ42 monomers compared to Aβ42 seeds. When high concentrations of Aβ40 seeds (0.5–1 μM, *i.e.* 25–50%) are added to Aβ42 monomers, an increase in ThT fluorescence is observed at an earlier time; however, the half-time is not shifted because the transition is less steep (Fig. 6E, F and M). High concentrations of Aβ40 seeds may thus affect the aggregation of Aβ42, but this effect lacks the strong auto-catalytic nature of the self-seeding reaction and may be due to unspecific effects that the addition of foreign material (in this case Aβ40 seeds) has on the aggregation reaction. When high concentrations of Aβ42 seeds are added to Aβ40 monomers an increase in ThT fluorescence is observed at an earlier time than for non-seeded samples; however, again the half-time is only marginally shifted compared to self-seeding (Fig. 6N). A more detailed discussion of possible reasons for this effect can be found in the ESI.† In all cases, the kinetics of cross-seeded samples much more closely resembles the kinetics of unseeded samples than the kinetics of self-seeded samples (Fig. 6). In the case of self-seeded kinetics data, the half-time shows a linear dependence on the logarithm of the seed concentration, in line with earlier findings.^41^ The cross-seeding data do not exhibit this feature but rather display a very flat relationship with slopes close to zero (Fig. 6M and N). These observations indicate that there is a very high level of sequence specificity in the surface-catalyzed nucleation and elongation processes. Processes B.ii/iii, C.ii/iii and D.i/ii in Fig. 1 are hence negligible.

The results of the seeding-experiments at pH 8.0 (Fig. S4†) differ from those conducted at pH 7.4. Both peptides display more efficient self-seeding at pH 8.0, and the data from cross-seeding experiments indicate a cross-seeding propensity which is significantly enhanced relative to the situation at pH 7.4. This efficacy is manifested as a shortened half-time for Aβ40 aggregation in the presence of Aβ42 seeds and *vice versa* (Fig. S4E†). This effect is also seen in the logarithm of the half-time *versus* log seed concentration, where the slope of the cross-seeding data deviates from zero at pH 8.0 (Fig. S4†) but not at pH 7.4 (Fig. 6).

### Monomer mixing does not affect secondary nucleation

The data obtained from the cross-seeding suggest that the presence of Aβ40 fibrils only has a minor, possibly non-specific, effect on the aggregation profile of Aβ42 and conversely. This is in contrast to the self-seeding effect observed for either of the two peptides, the aggregation of which is significantly catalyzed by the presence of fibrils formed from the same peptide. A major contribution to this effect is the catalysis of nucleation by the surfaces of fibrils, and the lack of this effect in cross-seeding experiments demonstrates the remarkable sequence selectivity of such a surface catalysis. A key question, however, is whether the surface catalytic effect can be restored through possible interactions of both monomeric forms of the peptides on a fibril surface (Fig. 1C.v/vi).

In order to investigate the effect of seed fibrils on monomer mixtures seeds of Aβ40 or Aβ42 were added to a 1 : 1 mixture of monomeric peptide (Fig. 7). Whereas cross-seeding simply tested for aggregation of nuclei of one species on the surface of fibrils of the other species, this setup determines whether this nucleation may be catalyzed by the presence of monomers of both species, *i.e.* the formation of co-nuclei on the fibril surface. Addition of preformed Aβ42 fibrils to an equimolar monomer mixture of Aβ42 and Aβ40 (1.5 μM + 1.5 μM) leads to acceleration of Aβ42 fibril formation seen as a shorter lag phase for the first transition while the second one is unaffected, Fig. 7A. This means that Aβ42 fibrils do not significantly catalyze the formation of co-nuclei which then go on to form Aβ40 fibrils (process C.v in Fig. 1). Adding seeds of Aβ40 leads to the opposite effect: only the lag phase for the second transition is shortened while the first one remains unaffected as shown in Fig. 7B, which indicates that Aβ40 fibrils have little effect on the formation of nuclei which then go on to form Aβ42 fibrils (process C.vi in Fig. 1). The analysis of the half-time of each transition as a function of seed concentration, Fig. 7C, shows that only Aβ42 seeds shorten the half-time for the Aβ42 assembly transition in the mixture. Conversely, the transition corresponding to Aβ40 fibril formation is only shortened by Aβ40 seeds. This shows that the formation of mixed nuclei on the surface of existing fibrils is negligible.

### Aggregation kinetics for Aβ42:Aβ40 monomer solutions

After having identified above through the use of mass spectrometry and *cryo*-TEM the first transition in mixed samples as Aβ42 aggregation and the second as Aβ40 aggregation, and having demonstrated that monomers mainly interact with fibrils of the same type, we can conclude that many of the processes that are theoretically possible in Fig. 1 are not major factors in the mixed aggregation of Aβ40:Aβ42 mixtures. In particular we can rule out a significant role for mixed elongation (B.ii/iii in Fig. 1) and mixed secondary nucleation (C.ii/iii/v/vi and D.i/ii in Fig. 1). As such, perturbations observed in the kinetics in a mixture of Aβ40 and Aβ42 must depend on the presence of the soluble rather than fibrillar forms (Fig. 1A). Taken together, the results from the seeding experiments show that cross-interactions between aggregated and monomeric forms of different species of Aβ only play a minor role for the aggregation reaction. We next set out to test whether interactions between Aβ peptides at the monomer level affect the kinetics of their aggregation. To this effect, we compared the effect of adding Aβ42 in either its monomeric or fibrillar forms to a solution of monomeric Aβ40 (Fig. 8). We observed that the changes in response to the addition of aggregated Aβ42 were very minor; by contrast, however, addition of monomeric Aβ42 to a solution of Aβ40 significantly accelerated the aggregation of Aβ40 (see also Fig. 9B and 10). Conversely the effect of monomeric Aβ40 on monomeric Aβ42 was negligible (see Fig. 9A and 10).

To further probe the origin of these interactions, we focused on the changes in the reaction half-time, in response to the presence of mixed Aβ40 and Aβ42 peptides in the solution (Fig. 9C). These data corroborate the finding that the aggregation of Aβ40 in the mixture is accelerated by Aβ42; the half-time is shorter for Aβ40 in the mixture compared to pure Aβ40. By contrast, fibril formation by Aβ42 in the mixture is affected in only a minor way by the presence of Aβ40. The solid lines (Fig. 9D) show a fit to a simple power laws (eqn (1)) describing the half-time as a function of peptide concentration. We find that the slopes in Fig. 9D are identical to within experimental error and therefore no significant difference in the exponent *γ* is found between pure Aβ42 and Aβ42 in the mixture, or between pure Aβ40 and Aβ40 in the mixture.

The exponent, *γ*, in such lag-time *versus* concentration plots is a good reporter on the reaction order of the dominant nucleation process; the fact that the slopes are similar therefore suggests that the nature, if not the rate, of the dominant nucleation mechanism in both Aβ40 and Aβ42 has remained unchanged in due to the presence of the other peptide relative to the situation when each peptide undergoes aggregation in pure solution (see also ESI and Fig. S7†). Moreover, the cross-seeding experiments suggest that both of the fiber-dependent molecular level processes, elongation and secondary nucleation, are largely unaffected. Thus, the changes in the lag-time can be ascribed to changes in the fiber-independent process, primary nucleation.

Finally, in order to confirm the large effect of Aβ42 on the aggregation on Aβ40, and the smaller effect of Aβ40 on the aggregation of Aβ42, we monitored a series of monomeric mixtures where the concentration of one of the peptides was maintained constant and that of the other was varied in a systematic manner. Fig. 9A and S5† show the results from an experiment where the concentration of Aβ42 is kept constant at 3 μM and the concentration of Aβ40 is varied, also leading to a variation in the total concentration of peptide. The converse data with the concentration of Aβ40 being maintained constant at 8 μM and the Aβ42 concentration being varied are shown in Fig. 9B. Only a small effect on the aggregation of Aβ42 is observed with increasing Aβ40 concentration, Fig. 9A. By contrast, the presence of Aβ42 accelerates strongly the aggregation of Aβ40, Fig. 9B. Because a relatively high Aβ40 concentration (8 μM) is used, the intermediate plateau is not as distinct under these conditions; however, the data nevertheless show a biphasic transition. The strong catalytic effect of Aβ42 on Aβ40 is furthermore evident in the data in Fig. 10 where high and low concentrations of Aβ40 are shown in separate panels, in all cases both pure and with 3 μM Aβ42. These data emphasize that the second transition observed in the mixed systems occurs earlier than the single transition in the corresponding pure sample with the same Aβ40 concentration.

To further illustrate these conclusions, simulated aggregation curves were generated (Fig. 11A) to probe whether changes in the primary pathway alone are sufficient to explain the experimental observation of the dramatic acceleration of Aβ40 aggregation by the presence of Aβ42 monomer in solution. First the aggregation of pure Aβ40 and Aβ42 were fitted using eqn (2). Then the aggregation of Aβ40 in the 1 : 1 mixture (second sigmoidal in Fig. 10C) was fitted allowing only the primary nucleation rate constant to change from the pure Aβ40 sample. The curve was fit well and the combined rate constant of primary nucleation in the 1 : 1 mixture was found to be approximately 2 orders of magnitude higher than in pure Aβ40. The simulated curve reproduces the characteristic double sigmoidal and demonstrates how an increase of the primary nucleation rate of Aβ40 alone can qualitatively reproduce the observed kinetics.

In summary the analysis shows that Aβ40 aggregation is affected strongly by Aβ42 monomers, but not Aβ42 fibrils, by promoting primary nucleation through interaction between the different monomeric species in solution. This mechanism is outlined in Fig. 11B. A particularly striking consequence of this mechanism is that although phase separation into distinct Aβ40 and Aβ42 fibrils is observed, mixed species may exist at the oligomer level and determine the rates of primary nucleation.

## Discussion

The results of this study show that separate fibrils are formed when Aβ42 and Aβ40 co-exist and reveal in molecular detail the aggregation process in binary mixtures. Although many studies have documented the mutual effect of Aβ42 and Aβ40 on their respective aggregation kinetics, this is to our knowledge the first time double transitions are observed in the overall aggregation curves starting from monomer mixtures, and the first time that the cross-reactivity is resolved over the composite steps in the aggregation mechanism.

These data are a remarkable manifestation of an extreme specificity in terms of which of the microscopic steps are amenable to modulation by the other peptide. While surface-catalyzed nucleation of monomers on fibril surface and elongation are highly specific events, there appears to be strong cooperation at the level of primary nucleation.

While a large number of studies have been devoted to aggregation processes in Aβ40:Aβ42 mixtures, we can only speculate over possible reasons why the double transition has not been observed before. The first and most likely reason is the high peptide concentrations used in previous studies. Our data from ThT fluorescence, CD and NMR spectroscopy, show that a clear separation between the two transitions is observed when the total and the relative peptide concentration are in the favorable range, below *ca.* 10 μM total peptide concentration. Another reason may be the use of synthetic peptide in many of the previous studies. Even with a high coupling efficiency (*α*) at each synthetic step, a 40 or 42 residue long peptide product will contain a major fraction (1 – *α* ^40^ or 1 – *α* ^42^) of peptides missing at least one residue at random; these peptides are very difficult to remove as they all have similar hydrophobicity and size as the full-length peptide and may co-elute during purification. A mixture of the two synthetic peptides will thus not be binary but contain in addition to Aβ42 and Aβ40, up to 42 different Aβ41, up to 1721 different Aβ40, a very large number of Aβ39 variants, *etc.* This may affect both the observed kinetics and the composition of the formed fibrils. Because of the high fidelity of the ribosome, the use of recombinant peptides provides superior sequence homogeneity. A third reason may be the use of co-solvents in some of the previous studies, which introduces one more component that can change the phase behavior of the system and affect the partition coefficients. A fourth reason may be a difference in starting state; which is clearly defined in a subset of the previous reports. In the present work, by taking control over every step from expression of sequence-homogeneous peptide, isolation of pure monomers, use of inert surfaces and controlled air–water interface, we observe very clearly double transitions in the aggregation process for Aβ42:Aβ40 monomer mixtures over a range of peptide concentrations. Moreover, the use of stable isotope labels in mass spectrometry and NMR spectroscopy unambiguously confirms separate aggregation events for Aβ42 and Aβ40.

While the present results imply that separate fibrils are formed in binary mixtures of Aβ42 and Aβ40, we cannot exclude a low level of incorporation of Aβ40 in Aβ42 fibrils or *vice versa*. A previous study at higher total peptide concentrations (10–24 μM), has indicated that Aβ40–Aβ42 monomer exchange may occur on fibrils.^30^ Loss of monomers by binding to the cognate fibrils might lead to reduced aggregation rate (see ESI†). Alternatively, the weak cross-seeding effect observed at high seed concentration may result simply from the increased surface area for nucleation, albeit in a form that lacks the very high catalytic activity observed in self-seeding.

**Fig. 12:**
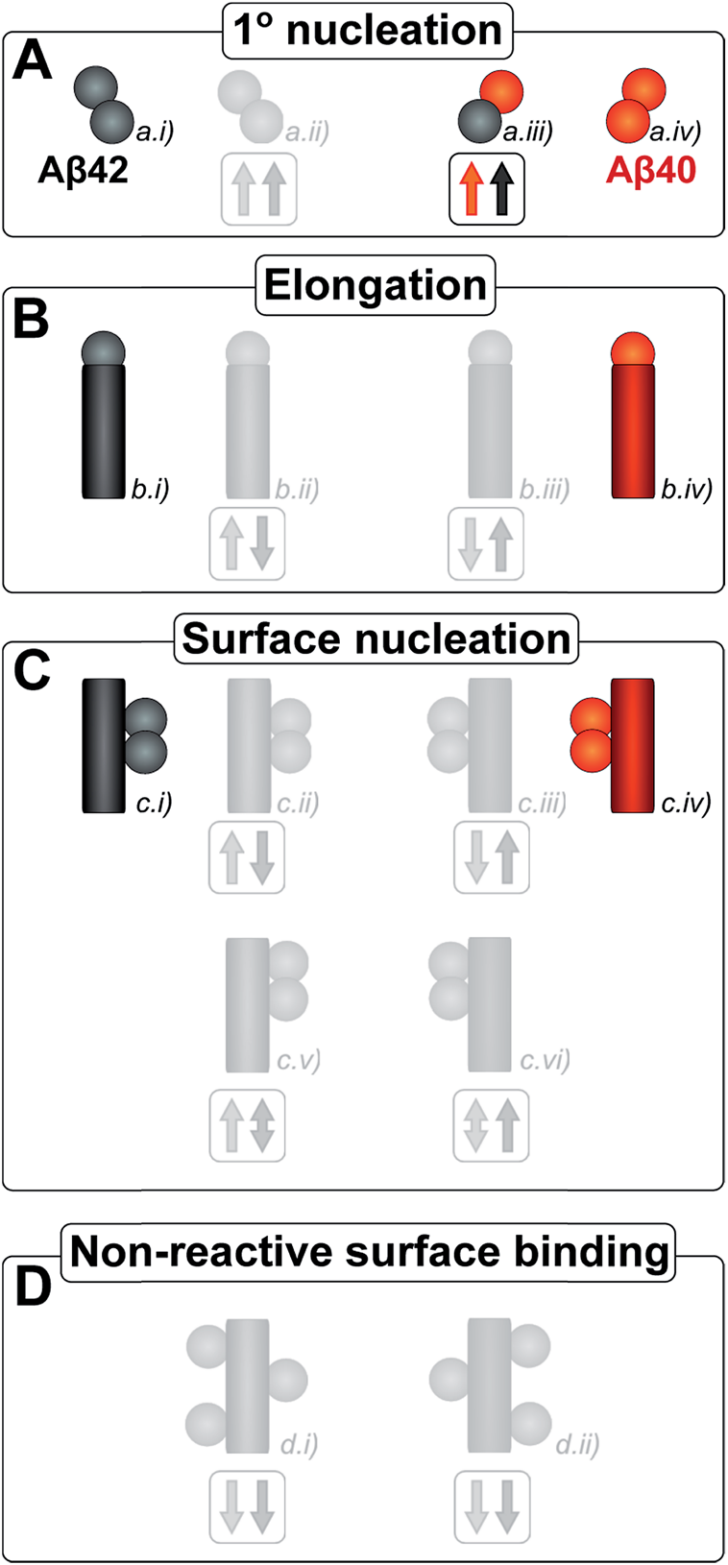
Microscopic reaction processes. Graphical depiction of the various simple reactions involving monomers or fibrils from either of the two protein species. Processes found to occur in a binary mixture of Aβ40 and Aβ42 peptides are shown in color. Processes that have been eliminated by results of the current work are shown in faint grey. Specifically the contributions to the Aβ42 primary nucleation (A.ii) could be neglected based on the aggregation from monomer mixtures, the cross elongation process (B.ii/iii) could be excluded from the absence of mixed fibrils and the surface nucleation (C.ii/iii & c.v/vi) and surface binding models (D.i/ii) were found to be negligible in the cross seeding reactions and seeding of mixtures.

After examining step by step the possibility for cross-reactivity in each microscopic process, we can erase several of the cross-reactions outlined in Fig. 1A–D and present a revised scheme (Fig. 12). In Fig. 11B, we present one model that is compatible with all the data in the current work and the mechanism for aggregation of Aβ40 (ref. 39) and Aβ42 (ref. 38) separately. The model is valid for macroscopic samples with multiple parallel processes.^11,47^ Here we studied samples of 10^14^ to 10^15^ monomers, which are well outside the stochastic regime and are therefore governed by rate constants and behave in a highly reproducible manner. Based on previous work on pure proteins the rate constant of primary nucleation is approximately two orders of magnitude higher for Aβ42 than for Aβ40.^39^ Therefore, at the beginning of the reaction the process is dominated by Aβ42 aggregation. However, some interaction at the level of primary nucleation clearly takes place since the second transition occurs earlier than for Aβ40 alone. This interaction between Aβ40 and Aβ42 peptides could manifest itself as the formation of relatively stable co-nuclei, or be of more transient nature, represented by the cloud in Fig. 11B. Whilst the presence of co-nuclei is certainly in agreement with our descriptions, it is not a necessary condition and transient interactions of the two peptides at the level of primary nucleation, rather than the formation of a stable mixed species, are also completely consistent with this description and all observed data. The cross-linking analysis (Fig. 2D) suggest that the concentration of co-nuclei is much lower than the concentration of Aβ42–42 nuclei. Co-nuclei may grow either by addition of Aβ42 or Aβ40 until they reach a size where further growth approaches the homogeneous rates (*k*_+42_ or *k*_+40_). The aggregates feed into their respective homomolecular autocatalytic cycles, the minor peptide component from the heterogeneous nucleation process may remain in the final aggregates, but will only be a minor contamination of the final fibrils.

Soon the majority of nuclei are formed from monomers in the highly specific surface-catalyzed secondary nucleation reactions rather than primary nucleation.^38,39^ From aggregation of pure peptides it has been established that as soon as 10 nM Aβ42 fibrils^38^ have formed, the majority of Aβ42 nuclei are formed by secondary rather than primary nucleation. For Aβ40 this effect is even more pronounced and secondary nucleation will be faster once 1 nM of Aβ40 fibrils has formed,^39^ making the aggregation of Aβ40 very sensitive to changes in primary nucleation.

The acceleration of Aβ40 by Aβ42 suggests that the rate of formation of Aβ40 fibrils *via* cooperation of the two peptides during heterogeneous primary nucleation is higher than the rate *via* homogeneous primary nucleation of Aβ40 alone. The lack of a large effect on the aggregation of Aβ42 by Aβ40 however suggests that the cooperation is negligible compared to the fast rate of primary nucleation from Aβ42 alone.

Hence the interaction during primary nucleation will serve to increase the Aβ40 fibril concentration in the early stages of the reaction quicker than in the pure case. This process then provides more catalytic surface for secondary nucleation of Aβ40 monomers on Aβ40 fibrils. Thus, more fibrils enter the autocatalytic feed-back loop for Aβ40 the higher the Aβ42 concentration and the overall process is accelerated even if only a small fraction of the Aβ40 monomers take the route of primary nucleation in cooperation with Aβ42. This cooperative nucleation happens at an intermediate rate and therefore has a significant effect on the slower process only, *i.e.* Aβ40 nucleation, explaining the observed asymmetry, where the presence of Aβ42 affects Aβ40 aggregation but not *vice versa*.

The high specificity of fibril dependent processes suggests a significant difference in the fibrillar state of Aβ40 and that of Aβ42, as might also be inferred from the large difference in ThT fluorescence intensity when bound to the fibrils and the large structural differences seen in cryo-TEM. Differences in the detailed packing of monomer units in the fibril propagate to distinct higher order structures and morphologies. To our knowledge, there is no high resolution X-ray structure of Aβ fibrils, but crystals formed from small amyloid-forming peptides reveal neat and highly repetitive packing of individual peptides.^20^ Crystals of peptide fragments from the C-terminal part of Aβ (Aβ35–40, Aβ35–42 and Aβ37–42)^48,49^ show well-ordered structures with a steric-zipper interface between β-sheets. Structural studies using solid state NMR and fiber diffraction have shown a number of structures for intact Aβ40 (ref. 50–54) and one for Aβ42.^55^ These structures show that the N-terminus is flexible and not involved in the inter-protofilament contacts. A major fraction of the residues, from *ca.* residue 11–17 to the C-terminus are tightly involved in the inter-β-strand interactions within each monomer that forms a β-turn-β topology and between monomers in the fibril.^50,55^ This may be the reason it is impossible to accommodate a two-residue C-terminal mismatch in the mature fibrils without significant increase in steric repulsion (Aβ42 in Aβ40 fibrils) or loss of favorable van der Waals interactions and hydrogen bonding. A less organized structure would better accommodate the two-residue C-terminal mismatch. This is likely the case for interactions at the monomer level such as in primary nuclei and some of the smaller oligomers, and therefore co-aggregation of Aβ40 and Aβ42 is tolerated at this level (Fig. 1A, and 11B).

Regardless of the detailed structure, the fact that separate fibrils are formed from mixtures allows us to draw some conclusions about the energetics of the reaction: assuming the end products of mixed fibrils are in equilibrium, our results imply that the free energy of pure fibrils is lower than for mixed fibrils. Hence the unfavorable entropy of de-mixing must be compensated by favorable interactions within the pure fibrils as it is unlikely that compensatory entropic contributions such as desolvation are much higher in the formation of pure fibrils compared to mixed fibrils. In other words the interactions of each peptide type with itself, within the fibrils, are significantly stronger than interactions between different types of peptide.

The extreme molecular specificity in the ability of fibrils to serve as surface-catalysts for nucleation is intriguing and provides insight into the molecular determinants of the homomolecular secondary nucleation process. Finally therefore, we lend ourselves to some speculation about the secondary nucleation reaction, which was recently discovered as a critical microscopic process in the aggregation mechanism for Aβ40 (ref. 39) as well as Aβ42,^38^ and underlies the near-exponential growth of the fibril concentration during the lag phase.^41^ However, little is known about structural aspects of the secondary nucleation *e.g.* in terms of where on the fibrils catalysis takes place, to which size(s) aggregates grow after nucleation, before detachment takes place, what the structure of the detached species is, and whether secondary and primary nuclei differ in structure or free energy. The failure of Aβ42 fibrils to catalyze nucleation of Aβ40 monomers, the failure of Aβ40 fibrils to catalyze nucleation of Aβ42 monomers, and the failure of each fibril to catalyze the formation of Aβ42–Aβ40 co-nuclei, implies that a general surface effect is not enough to explain the highly efficient surface catalyzed secondary nucleation in each pure case. In contrast, nucleation of Aβ40 or Aβ42 can be catalyzed by foreign surfaces, such as for example nanoparticles^56,57^ or positively charged polymers,^58^ which presumably lack any kind of structural complementarity to Aβ, this effect may be explained by surface attraction (locally enriched Aβ concentration) promoting primary nucleation to proceed faster than in bulk.^58^ The current findings of extreme specificity in fibril-catalyzed nucleation suggest that the attraction between Aβ peptides and dislike fibrils is too low for such general surface effect to arise, presumably due to significant electrostatic repulsion; each monomer has a net charge between –3 and –4 and the fibril carries a considerable negative surface charge. This would imply that in the homomolecular secondary nucleation reaction, nucleation is favored by some structural complementarity between the surface and the nucleating peptides, which overcomes this repulsion. Possibly, the incoming monomers engage on the fibril surface to form a pre-nucleus that copies the low free-energy structure of seed fibril in a manner which generates stable enough species that after detachment grow faster by further monomer addition compared to their dissociation back into monomers. The failure of Aβ42 to nucleate on Aβ40 fibril, and *vice versa*, might reflect that considerable steric repulsion, or other unfavorable interactions, would occur if Aβ42 copied the structure of Aβ peptides in the Aβ40 fibril leading to a highly unstable, disfavored and improbable transition state.§
§Note added in proof: After this paper was accepted, one new model of Aβ42 fibrils based on solid-state NMR was reported together with one experiment showing that self-seeding of Aβ40 with 20% Aβ40 fibrils is much more effective than cross-seeding with 20% Aβ42 fibrils (ref. 62).


## Conclusions

Aβ40 and Aβ42 interact strongly at the level of primary nucleation, but only the smallest aggregates may exist as mixed species. This perturbation of primary nucleation leads to a significant acceleration of Aβ40 aggregation in the presence of Aβ42 monomers. However, fibril elongation and surface-catalyzed secondary nucleation are highly specific events resulting in the formation of distinct fibrils composed of Aβ42 or Aβ40. Thus comparing the stability of pure to mixed fibrils, in pure fibrils the entropic de-mixing penalty is compensated by much more favorable interactions within the fibril. The total as well as relative peptide concentration determines whether a single or double transition is observed for a process that starts from an Aβ40/Aβ42 monomer mixture. At low total concentrations (below *ca.* 10 μM) two transitions are seen while a single transition is observed at higher total concentrations. The observation of cross-reactivity exclusively at the level of primary nucleation implies that this reaction is least discriminative among the microscopic steps that underlie the amyloid formation reaction, reflecting that primary nuclei, and possibly also some of the small oligomeric species, have the lowest level of structural organization among all the species in the amyloid formation reaction.

## Methods

### Expression and purification of peptides

The genes coding for wild type Aβ(M1-42), here called Aβ42, Aβ(M1-40), here called Aβ40, were produced by overlapping PCR, cloned into the PetSac vector and expressed in *E. coli*. ^14^N-peptides were expressed in rich medium. ^15^N-Aβ42 and ^13^C^15^N-Aβ42 were expressed in M9 minimal medium with ^15^NH_4_Cl as the nitrogen source and ^13^C-glucose as the carbon source. The peptides were purified using sonication, isolation of inclusion bodies by centrifugation, dissolving the inclusion bodies in urea, and isolation of the peptide by ion exchange and size exclusion steps as described.^59^ Purified peptide aliquots were lyophilized and stored as dried powder until use.

### Preparation of samples for kinetic experiments

Each experiment started with a size exclusion chromatography (SEC) step to isolate monomeric peptide. Approximately 50–350 μg of peptide was dissolved in 1.0 mL 6 M GuHCl for 30 min (to dissolve pre-existing aggregates) and injected onto a Superdex 75 10/300 GL column using a fast protein liquid chromatography (FPLC) system and eluted at 0.7 mL min^–1^ in the desired buffer (Fig. S6†). For ThT and *cryo*-TEM experiments, monomers were eluted in 20 mM sodium phosphate buffer, pH 7.4 or 8.0, with 200 μM EDTA and 0.02% NaN_3_, whereas 5 mM sodium phosphate with 40 mM NaF, pH 7.4 or 8.0, was used for CD spectroscopy. The center of the monomer peak was collected (Fig. S6†) to minimize contamination from *E. coli* proteins or soluble aggregates or salts. The peptide concentration was determined by integrating the absorbance at 280 nm of the collected peak using *ε*_280_ = 1440 M^–1^ cm^–1^ and by amino acid analysis after acid hydrolysis (purchased from BMC, Uppsala).

### Aggregation kinetics experiments

Both the monomer solution and the sodium phosphate buffer used for preparing the experimental samples were supplemented with 5 μM thioflavin T (ThT) from a concentrated stock. All solutions were kept on ice before starting the experiments. Low binding Eppendorf tubes (Genuine Axygen Quality, Microtubes, MCT-200-L-C) were used to prepare samples with concentrations ranging between 1 and 20 μM. Each tube contained a total volume of 300–400 μL and the solution was mixed by gently turning the tube upside down, instead of vortexing, to avoid air bubbles. A Corning 3881, 96 well half-area plate of black polystyrene with clear bottom and PEG coating was used and each well was loaded with 100 μL sample. The samples with lowest overall aggregation rate were loaded first and those with the highest last. The plate was sealed with a plastic film (Corning 3095). The plate was placed in a Fluostar Omega or Fluostar Optima plate reader (BMG Labtech, Offenburg, Germany) and incubated at 37 °C in quiescent condition between reads. ThT fluorescence was measured at different time intervals for up to 72 h through the bottom of the plate, with excitation and emission wavelengths of 440 nm and 480 nm, respectively. Each experiment was set up at least twice in different plates with quadruplicate replicates of each sample. The half-time (*t*_1/2_) of the aggregation process was estimated by finding the time point at which the ThT fluorescence was half way in between the starting and ending baselines of the transition.

The following power function was used to fit the half-time *versus* the total Aβ concentration, *c*

1 *t*_1/2_(*c*) = *Bc*^*γ*^

where *B* is a proportionality constant and *γ* is the exponent. The fibrils used in the seeding experiments were collected immediately after reaching the ThT plateau, then stored in a low-binding Eppendorf-tube on ice and used within a few hours. In previous studies no difference in kinetics was noticed between samples supplemented with freshly prepared fibrils that were sonicated for 0, 2 or 10 min in a sonicator bath.^41^ This shows that the parameter that matters for secondary nucleation which dominates at low seed concentrations is the surface area, not the number of fibril ends.

### Cryogenic transmission electron microscopy (*cryo*-TEM)

The samples used for *cryo*-TEM were prepared and incubated in the same way as for the kinetic aggregation studies. Samples with 10 μM of Aβ40, Aβ42, or 1 : 1 mixtures of Aβ42 : Aβ40 (5 μM of each peptide in the mixtures) were monitored by ThT and collected at the plateau (both first and second plateau for the Aβ42:Aβ40 mixture) and kept at 4 °C (maximum time kept at 4 °C was over night) until imaged by cryo-TEM. Specimens for electron microscopy were prepared in a controlled environment vitrification system (CEVS) to ensure stable temperature and to avoid loss of solution during sample preparation. The specimens were prepared as thin liquid films, <300 nm thick, on lacey carbon filmed copper grids and plunged into liquid ethane at –180 °C. This leads to vitrified specimens, avoiding component segmentation and rearrangement, and water crystallization, thereby preserving original microstructures. The vitrified specimens were stored under liquid nitrogen until measured. An Oxford CT3500 cryoholder and its workstation were used to transfer the specimen into the electron microscope (Philips CM120 BioTWIN Cryo) equipped with a post-column energy filter (Gatan GIF100). The acceleration voltage was 120 kV. The images were recorded digitally with a CCD camera under low electron dose conditions. The node-to-node distance was measured using the Digital Graph software (Gatan, Inc.).

### Circular dichroism (CD) spectroscopy

The ellipticity was recorded between 250 nm and 190 nm in a quartz (QS) cuvette with 10 mm path length at 37 °C during continuous stirring to avoid sedimentation of fibrils below the measurement zone, using a Jasco J-815 CD spectrometer. The scanning rate was 50 nm min^–1^, the digital integration time per data point (D.I.T.) 8 s, sensitivity set to standard, the background signal from the buffer has been subtracted and the reported data are averaged over 3 accumulations. 5 mM sodium phosphate buffer, pH 7.4 or 8.0 with 40 mM NaF was used and the total peptide concentration 5 μM or 10 μM.

### Monomer depletion in an equimolar mixture of Aβ42 and Aβ40 by mass spectrometry (MS)

The samples used for MS were prepared and incubated in the same way as for the kinetic aggregation studies except that ^15^N-Aβ42 was used to allow distinction from Aβ40 after tryptic digestion. Samples were prepared to initially contain ^15^N-Aβ42 and ^14^N-Aβ40 monomers at 1 : 1 ratio at 3, 5 or 10 μM total concentration. All samples contained 5 μM ThT. Multiple identical 100 μL samples were placed in wells of a 96-well plate (Corning 3881) which was incubated at 37 °C. Samples (100 μL) were withdrawn from wells at start and at different time points during the two transitions and at the first and second plateau. In addition to the withdrawn samples, quadruplicates of each solution were kept untouched in the plate and the ThT fluorescence monitored at 37 °C. The withdrawn samples were centrifuged at 20 000*g* for 5 min at r.t. to pellet aggregates and 15 μL of the supernatant transferred to another low binding Eppendorf tube on ice containing trypsin (sequencing grade, Promega, Sunnyvale, CA) at 0.02 molar ratio. The samples were incubated for 16 h at 37 °C and the digestion was stopped by adding 1.6 μL 1% TFA. Samples were stored in the freezer (–20 °C) prior to analysis. Samples were dried under vacuum and dissolved in 4 μL 0.1% TFA, 1% ACN. 0.5 μL samples were dispensed onto a MALDI sample support and allowed to air-dry prior to addition of matrix solution (4-hydroxy-α-cyano cinnamic acid in 60% acetonitrile, 0.1% TFA). MALDI-TOF mass spectrometry was performed using a 4700 proteomics analyzer (Applied Biosystems, Framingham, MA). All analyses were performed in positive reflector mode collecting data from approximately 3000 single laser shots. The ratio of Aβ42/Aβ40 monomer at different time points was calculated from the relative intensities of the 777.25 and 768.28 peaks representing ^15^N-Aβ(M1-5) and ^14^N-Aβ(M1-5), respectively.

### Identification of fibrils in an equimolar mixture of Aβ42 and Aβ40 by mass spectrometry (MS)

Samples were prepared as described in the section above to initially contain ^15^N-Aβ42 and ^14^N-Aβ40 monomers at 1 : 1 ratio at 3 μM total concentration. All samples contained 5 μM ThT.

Samples (300 μL) were withdrawn at the first and second plateau and filtrated through 0.2 μm spin filter (VIVASPIN 500, Sartorius Stedim Biotech) to trap the fibrils. Fibrils trapped on the filter were washed with 10 times volume of Milli-Q water to remove any small aggregates and the retentate volume for each sample was *ca.* 50 μL. The fibrils were digested, stored, dried and dissolved as described above. In addition the samples were desalted using C_18_ tips (Pierce Protein Biology Product, Thermo Scientific) prior to analysis. All analysis was performed as described above.

### Identification of cross-linked peptides in an equimolar mixture of Aβ42 and Aβ40 by mass spectrometry (MS)

Samples were prepared as described in the MS experiments above at 1 : 1 ratio at 3 μM total concentration. Three samples containing 5 μM ThT were used to follow the aggregation kinetics as described in the aggregation kinetic experiments above. Eight samples were incubated at 37 °C without ThT in low binding Eppendorf tubes (Genuine Axygen Quality, Microtubes, MCT-200-L-C). Cross-linking was initiated after 0, 10, 20, 30, 40, 50, 60 and 70 min by adding 3 μL 100 μM cross-linker BS3 (Pierce Protein Biology Products, Thermo Scientific) that was dissolved in Milli-Q water and incubated at room temperature for 1 min. The reaction was quenched after 1 minute incubation at room temperature by adding 1 μL 0.5 M Tris followed by placing the sample on ice for 5 min. The samples were digested with trypsin, stored, dried, dissolved and analyzed as described above.

### Nuclear magnetic resonance (NMR) spectroscopy

The aggregation time courses starting from equimolar mixtures of ^13^C-Aβ42 and ^12^C-Aβ40 monomers (total concentration 5, 10 or 20 μM) were monitored at 37 °C, pH 7.4 at a magnetic field strength of 14.1 T. A series of 1D proton spectra were alternatingly acquired with selection or filtering of ^13^C bound protons (optimized for methyl groups), 3 min acquisition for each spectrum. Spectra were processed with NMRPipe^60^ and methyl group signals integrated in Matlab. Spectra of isolated ^13^C-Aβ42 and ^12^C-Aβ40 monomers were acquired under the same conditions. For ^12^C-Aβ40 no signal of ^13^C bound protons could be detected in the selection experiment. In contrast for ^13^C-Aβ42 a residual signal from ^12^C bound protons could be observed in the ^13^C filtered experiment. This was accounted for in the filtered spectra of the kinetic experiment.

### Theoretical analyses

The integrated rate law for filament growth under the action of primary and secondary nucleation coupled to elongation is given in closed form as:2
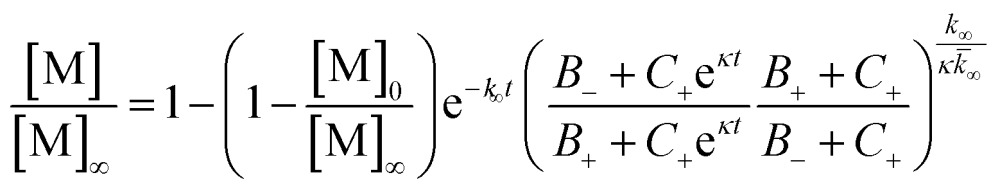
where the parameters are defined by
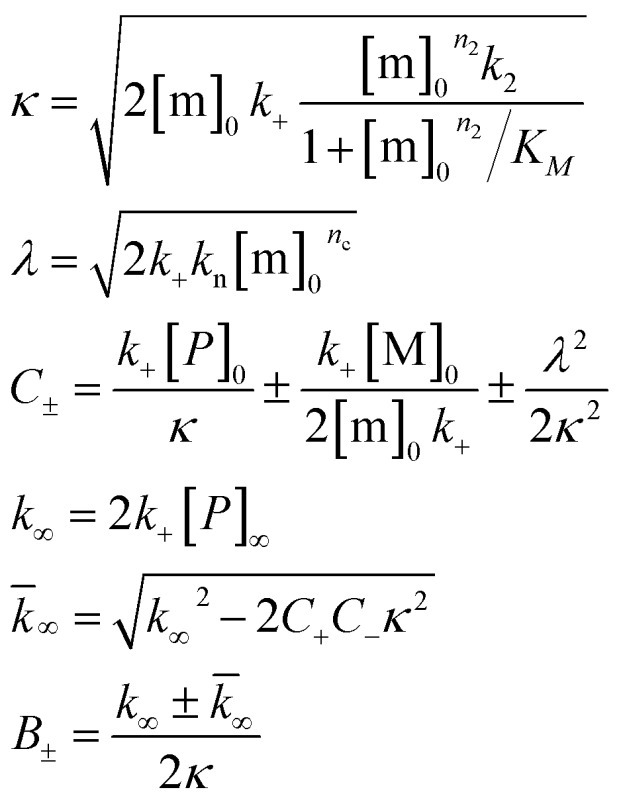
 
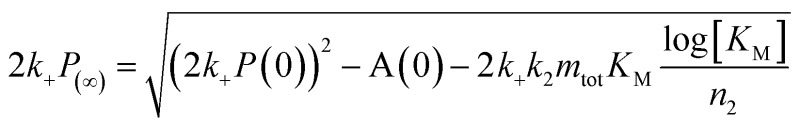
[m]_0_ is the initial monomer concentration and [*P*]_0_, [M]_0_ and [*P*]_∞_, [M]_∞_ are the aggregate number and mass concentration at the start of the reaction and in equilibrium. *k*_+_, *k*_n_ and *k*_2_ are the rate constants of elongation, primary nucleation and secondary nucleation respectively. *K*_M_ is the saturation constant for secondary nucleation. *n*_2_ and *n*_c_ are the monomer scalings of primary and secondary nucleation. *n*_c_ = *n*_2_ = 2 was used in all cases as previously found for pure Aβ42 and pure Aβ40. Co-nuclei are likely to have reaction orders comparable to those of the nuclei formed of pure peptide (see ESI†). The global fitting to this equation was performed using a fitting algorithm based on the principle of basin hopping.^61^
