# Supplementary material for: The Aβ40 and Aβ42 peptides self-assemble into separate homomolecular fibrils in binary mixtures but cross-react during primary nucleation
Source: Chem Sci. 2015 May 8;6(7):4215–33. doi: 10.1039/c4sc02517b (PMC5707514; doi:10.1039/c4sc02517b)
Supplement: Supplementary file 1 [file SC-006-C4SC02517B-s001.pdf]

Electronic Supplementary Material (ESI) for Chemical Science.
This journal is © The Royal Society of Chemistry 2015

The Aβ40 and Aβ42 peptides self-assemble into separate homomolecular fibrils in binary mixtures but cross-react during primary nucleation

Risto Cukalevski[1]*, Xiaoting Yang[1]*, Georg Meisl[2], Ulrich Weininger[3], Birgitta Frohm[1], Tuomas P.J. Knowles[2], Sara Linse[1]
1. Lund University, Biochemistry and Structural Biology, Chemical Centre, Lund, Sweden
2. Cambridge University, Chemistry Department, Lensfield Road, Cambridge, UK.
3. Department of Biophysical Chemistry, Center for Molecular Protein Science, Lund University, Lund, Sweden
*contributed equally

Supporting Information

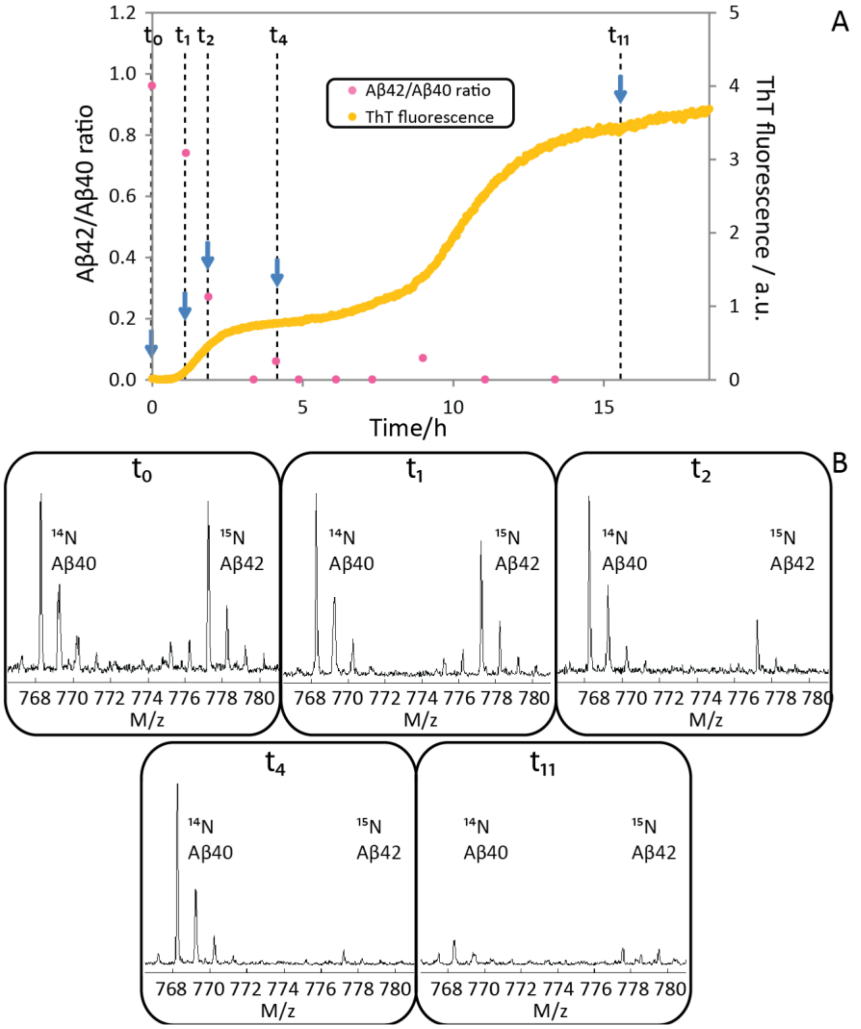

Fig. S1 Aggregation in Aβ42:Aβ40 monomer mixtures as a function of time. A) ThT fluorescence (yellow), and the ratio of Aβ42/Aβ40 monomer concentration remaining in solution at 11 time points measured by mass spectrometry (pink). The mixture contains 2.5 µM Aβ42 and 2.5 µM Aβ40. The process displays two transitions by ThT fluorescence. **B)** The corresponding mass spectra from five of the time points (indicated by arrows in panel A). The Aβ42/Aβ40 ratio is close to 1 at t_0 and decreases to close to 0 after the first plateau has been reached. Aβ42 monomer is depleted during the first sigmoidal transition and Aβ40 monomer is consumed during the second sigmoidal transition, suggesting the formation of separate fibrils. The samples contain 5 µM ThT, 20 mM sodium phosphate, 200 µM EDTA, 0.02% NaN_3, pH 7.4.

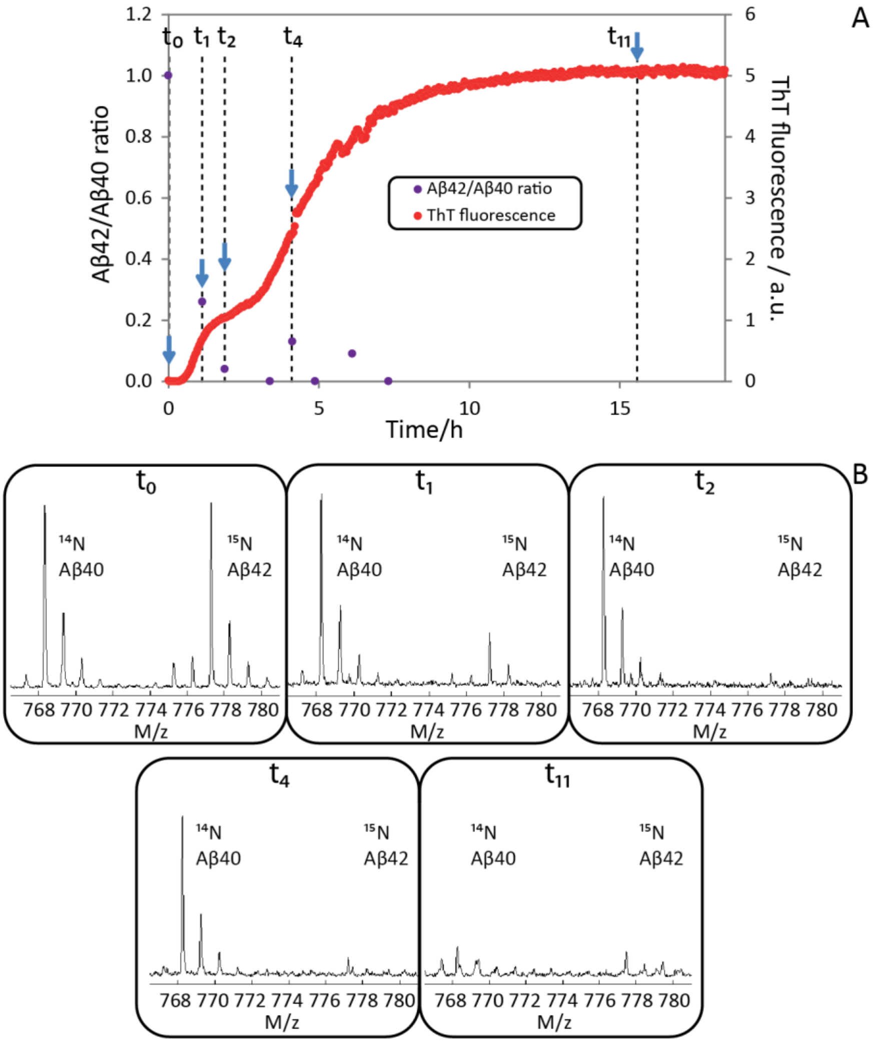

Fig. S2 Aggregation in Aβ42:Aβ40 monomer mixtures as a function of time. A) ThT fluorescence (red), and the ratio of Aβ42/Aβ40 monomer concentration remaining in solution at 8 time points measured by mass spectrometry (purple). Mixture contains 5 µM Aβ42 and 5 µM Aβ40. The process displays two transitions by ThT fluorescence. **B)** The corresponding mass spectra from five of the time points (indicated by arrows in panel **A)**. The Aβ42/Aβ40 ratio is close to 1 at t_0 and decreases to close to 0 after the first plateau has been reached. Aβ42 monomer is depleted during the first sigmoidal transition and Aβ40 monomer is consumed during the second sigmoidal transition, suggesting the formation of separate fibrils. The samples contain 5 µM ThT, 20 mM sodium phosphate, 200 µM EDTA, 0.02% NaN_3, pH 7.4.

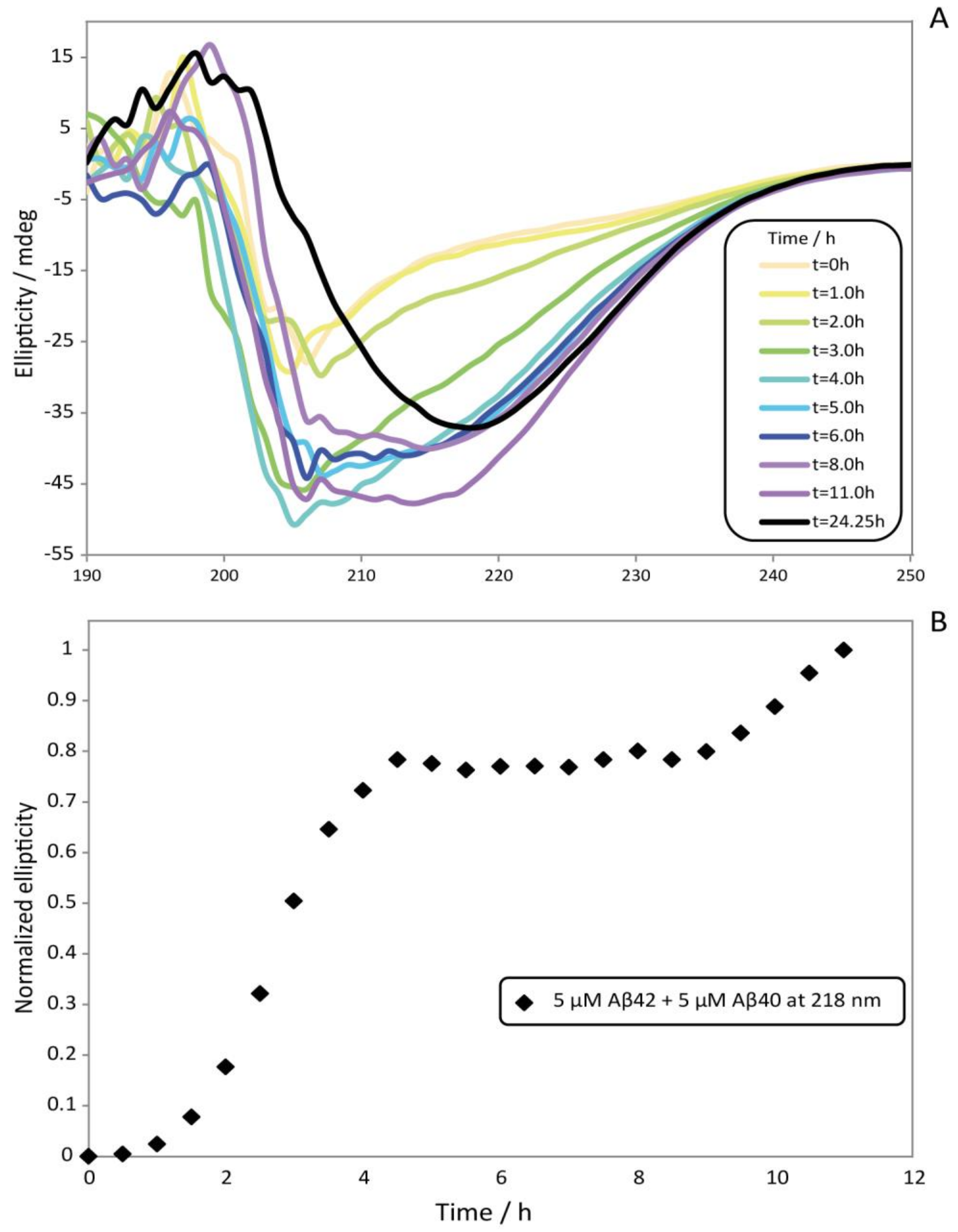

Fig. S3 Secondary structural change monitored by far-UV CD spectroscopy for a mixture of 5 µM Aβ42 and 5 µM Aβ40. A) Far-UV CD spectra as a function of time monitor the transition from random coil to β-sheet. The minima shifts from around 202 nm at time zero to 210 nm after 4 h and 7 h later it shifts to 218 nm. After 24 h of incubation the ellipticity increases due to the precipitation of the amyloid fibrils and the minima is around 218 nm. **B)** Normalized ellipticity at 218 nm as a function of time. The ellipticity increases during the first 5 h and displays a sigmoidal curve which probably is due to Aβ42 forming fibrils. After 9 h the ellipticity increases again indicating that the second transition has started and that Aβ40 starts to form fibrils. The sample contains 5 mM sodium phosphate and 40 mM NaF, pH 8.0.

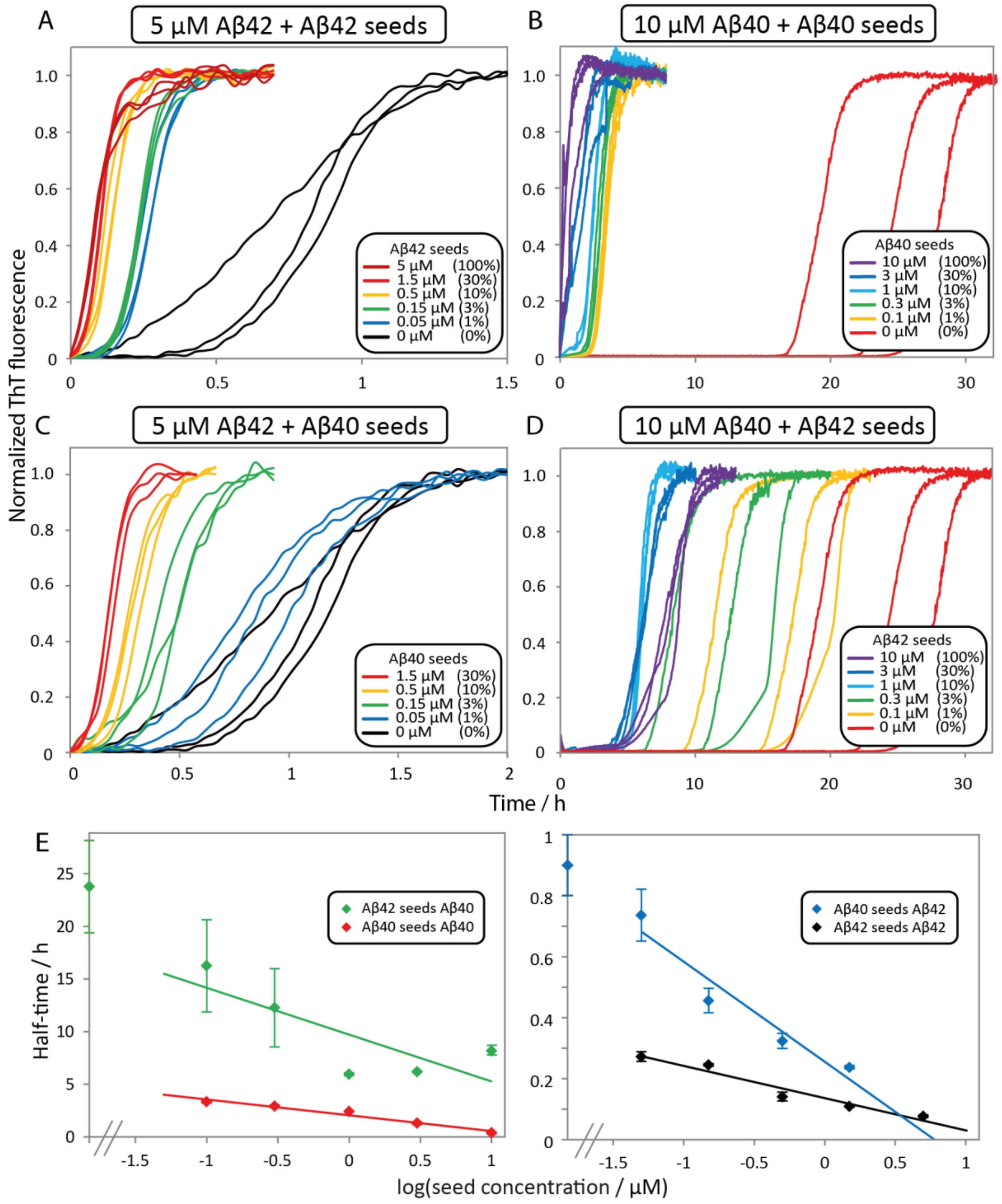

Fig. S4 Self and cross-seeding experiment of Aβ42 and Aβ40. A) and **B)** Self-seeding of Aβ42 and Aβ40, respectively. With increasing seed concentration the lag-phase decreases until the sigmoidal shape disappears. **C)** Aβ42 monomer seeded by Aβ40 fibrils. **D)** Aβ40 monomer seeded by Aβ42 fibrils. **E)** Half-time as a function of the logarithm of the seed concentration. Both self-seeding and cross-seeding data displays clear concentration dependence though the self-seeding is more efficient. Each data point is an average of at least three replicates with error bars representing the standard deviation. The samples contain 5 µM ThT, 20 mM sodium phosphate, 200 µM EDTA, 0.02% NaN_3, pH 8.0.

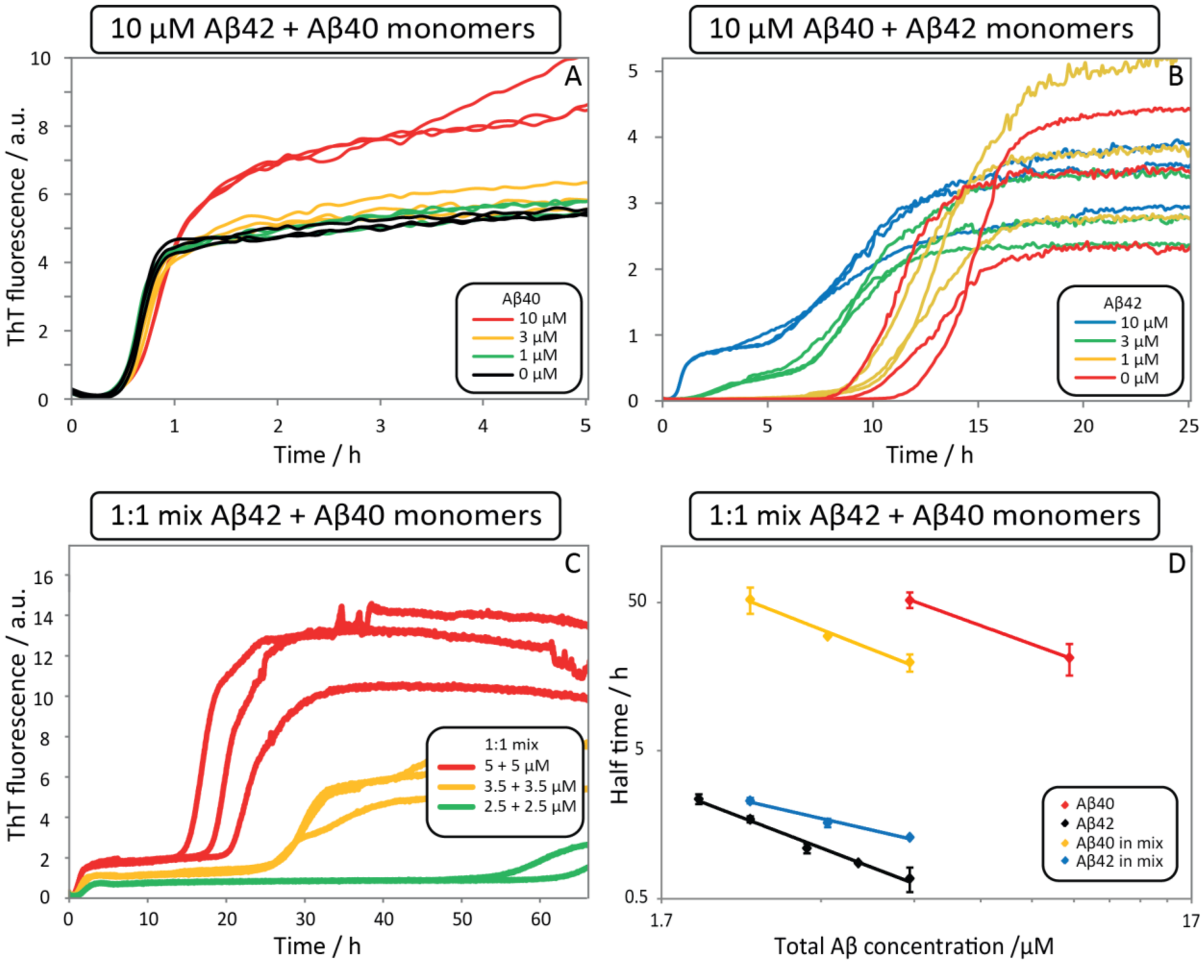

Fig. S5 Aggregation kinetics for mixtures of Aβ42 and Aβ40, and of pure peptides. ThT fluorescence intensity as a function of time. **A)** 10 µM Aβ42 and varied Aβ40 concentrations. The effect of Aβ40 on Aβ42 is small. **B)** 10 µM Aβ40 and varied Aβ42 concentrations. Aβ40 is accelerated significantly by Aβ42, seen by a shorter lag-phase of the second transition as Aβ42 concentration is increased. **C)** ThT fluorescence intensity as a function of time for equimolar mixtures at three different concentrations. Three replicates of each concentration are shown. **D)** The half-time of the kinetics in C as a function of total Aβ concentration. The half-time is defined as the point in time where the ThT fluorescence is half-way between baseline and first plateau values for Aβ42 or first and second plateau values for Aβ40. Each point is an average of three replicates with error bars representing the standard deviation. The solid lines are power functions fitted to the experimental data. Aβ40 aggregation in the mixture is accelerated by Aβ42 seen by a shorter half-time (compare red with yellow) while Aβ42 aggregation is not affected significantly by Aβ40 (compare black with blue). All samples contain 5 µM ThT, 20 mM sodium phosphate, 200 µM EDTA, 0.02% NaN_3, pH 8.0.

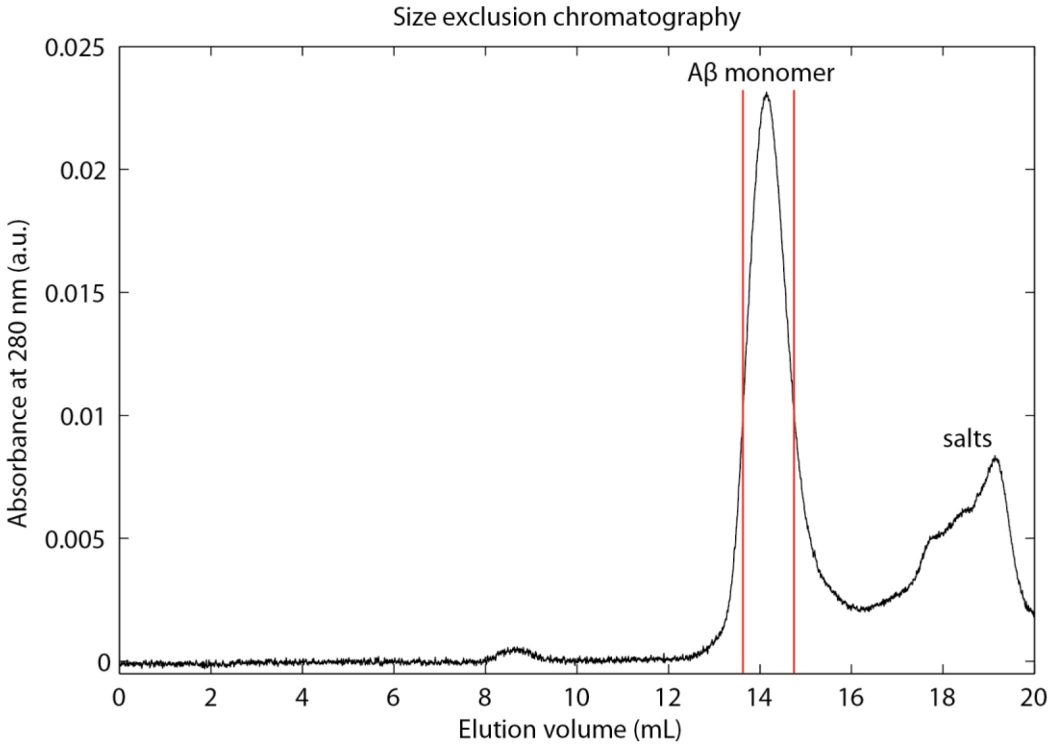

Fig. S6 Isolation of the monomer by size exclusion chromatography (SEC) on a fast protein liquid chromatography (FPLC) system using a Superdex 75 10/300 GL column. A narrow fraction of the monomer peak is collected (red lines) to minimize contamination from *E.coli* proteins, soluble aggregates or salts (mainly from Tris, EDTA and GuHCl) from previous purification step.

Estimating the reaction orders of the formation of mixed primary nuclei.

The rate of formation of nuclei that can elongate via addition of Aβ40, through the process of primary nucleation, is given by

$$\frac{dP}{dt} = k_{40,\mathrm{pure}} m_{40}^{n_{40,\mathrm{pure}}} + k_{40,\mathrm{mix}} m_{40}^{n_{40,\mathrm{mix}}} m_{42}^{n_{42,\mathrm{mix}}} \qquad (1)$$

where the *k* denote the rate constants of primary nucleation of pure and mixed nuclei and the *n* denote the scaling exponents of Aβ40 and Aβ42 in each of these processes.
The scaling exponent for the formation of pure nuclei where determined in previous work[38,39], so here we attempt to determine the scaling exponents of mixed nucleus formation, $n_{40,mix}$ and $n_{42,mix}$.

First consider the aggregation of a constant concentration of Aβ40 in the presence of varying concentrations of Aβ42. According to the model we propose, the only significant effect of

Aβ42 addition is the formation of co-nuclei, as captured by the second term in equation 1. We now aim to obtain an expression for the half time of aggregation of Aβ40 as a function of the concentration of Aβ42 in order to determine $n_{42,mix}$.

For an unseeded aggregation reaction of pre protein the half time is given by:

$$t_{1/2} = -\frac{\log(C_+)}{\kappa} + A \qquad (2)$$

where A is a constant and

$$C_+ = \lambda^2/(2\kappa^2) \text{ with}$$

$$\kappa = \sqrt{2k_+k_2m_0^{n_2+1}}$$

$$\lambda = \sqrt{2k_+k_nm_0^{n_c}}$$

In order to include the rate of formation of new nuclei from co-oligomers, we need to replace the term

$$k_nm_0^{n_c}$$

by

$$k_{\text{pure}}m_{40}^{n_{40,\text{pure}}} + k_{\text{mix}}m_{40}^{n_{40,\text{mix}}}m_{42}^{n_{42,\text{mix}}}.$$

The half time then becomes:

$$\begin{aligned} t_{1/2} &= -\frac{\log(\frac{k_{\text{pure}}}{k_2}m_{40}^{n_{40,\text{pure}}-n_2-1} + \frac{k_{\text{mix}}}{k_2}m_{40}^{n_{40,\text{mix}}-n_2-1}m_{42}^{n_{42,\text{mix}}})}{\kappa} + A \\ &= -\frac{\log(1 + \frac{k_{\text{mix}}}{k_{\text{pure}}}m_{40}^{n_{40,\text{mix}}-n_{40,\text{pure}}}m_{42}^{n_{42,\text{mix}}})}{\kappa} + \bar{A} \\ &= -\frac{\log(1+\alpha m_{42}^{n_{42,\text{mix}}})}{\kappa} + \bar{A} \qquad (4) \end{aligned}$$

where on the second line an Aβ42 concentration independent factor was absorbed into the constant $\bar{A}$ and on the last line the term

$$\frac{k_{\text{mix}}}{k_{\text{pure}}}m_{40}^{n_{40,\text{mix}}-n_{40,\text{pure}}}$$

was replaced with α for clarity. As κ can be determined from the aggregation of pure Aβ40 as 0.4 h^{-1} (reference 40), equation 4 can be fitted with 3 free parameters, $\bar{A}$, α and the scaling exponent $n_{42,mix}$. Fitting to the half time of aggregation of Aβ40 in the presence of varying concentrations of Aβ42 (aggregation data shown in Figure 9B, fit shown in Figure S7 below, yields a low scaling exponent $n_{42,mix}$ of approximately 0.5. In general, a scaling exponent below 1 suggest a multistep reaction mechanism, where one of the steps is independent of Aβ40 concentration. However, this value needs to be interpreted with caution as it is likely to be only approximate, due to errors in the determination of κ, errors in the determination of the half times (due to the difficulty of distinguishing the ThT signal from Aβ40 and Aβ42 at

concentrations where the double sigmoidals show little separation) and the underlying assumption that co-nuclei elongate at the same rate as pure nuclei. Nonetheless the low value of the scaling suggests that co nuclei are likely to be very similar in size to the nuclei found in the aggregation of pure protein.

An estimate of the scaling exponent of Aβ40, $n_{40,mix}$, is much harder to obtain. The equivalent method of considering the effect of a varying Aβ40 on Aβ42 aggregation at a constant Aβ42 concentration is not useful because the aggregation of Aβ42 is not significantly affected by Aβ40 (most likely due to the rate of mixed nucleus formation being negligible compared to the rate of formation of pure Aβ42 nuclei. This would be reflected by a very small α in equation 4.) The other option would be to consider the aggregation of Aβ40 in the presence of a constant concentration of Aβ42. However, the variation in Aβ40 concentration also affects the secondary processes, which contribute to the half time directly, via the parameter κ, whereas the contribution of the primary nucleation process enters only as a logarithmic correction. Therefore any effects on primary nucleation are likely to be a negligible contribution to the half time, this approach also unfeasible.

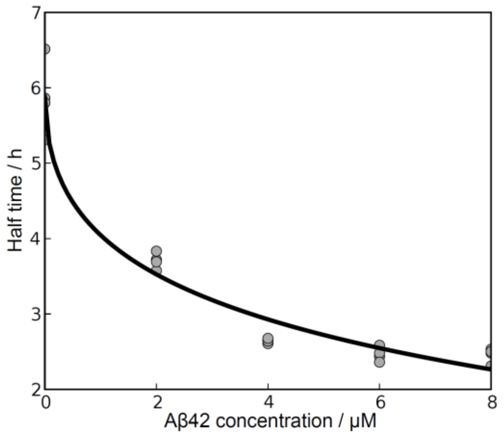

Figure S7. The half time of Aβ40 aggregation as a function of Aβ42 concentration. The solid line represents a fit of equation 4 with three free parameters: α, $\bar{A}$ and $n_{42,mix}$.

Effect of cross seeding at high concentrations

Aβ40 seeded with Aβ42 fibrils: The effect of Aβ42 seeds on the aggregation of Aβ40 is very small compared to the effect of self seeding. The slight decrease in lag time with increasing seed is most likely the result of introducing a heterogeneous surface (the Aβ42 seeds) that promotes primary nucleation, rather than any specific effect of Aβ42 seeds. Aβ aggregation has been shown to strongly depend on surface effects[56,57].

Aβ42 seeded with Aβ40 fibrils: The effect of Aβ40 seeds on the aggregation of Aβ42 is slightly more pronounced. It is worth noting, however, that this situation (large amounts of fibrillar Aβ40 in the presence of monomeric Aβ42) will not occur in aggregation from mixed monomers as Aβ42 aggregation is much faster and will from fibrils before Aβ40 can.

Several mechanisms by which seeds of one type affect the aggregation of monomers of another type can be imagined. The simplest ones are considered here:

- Seeds may catalyze nucleus formation of the other peptide on their surface. This is simply heterogeneous nucleation and will give an additional term similar to primary nucleation, i.e. lead to an effective increase in k_n.

- Seeds may elongate with monomers from the other peptide. In the equations this can be simulated by having a non zero initial fibril number P(0) but no initial fibril mass M(0)=0.

- Seeds may sequester monomer, effectively removing it from the reaction. In the equations this will be reflected by having a lower initial monomer concentration m(0). In contrast to the other mechanisms mentioned above this is expected to slow the aggregation as the seed concentration increases.

The fact that the half-time of each peptide remains largely unchanged as the concentration of seeds of the other peptide increases, although the curve shapes change, suggests the presence of two opposing effects that lead on the one hand to shorter lag times but on the other hand to shallower curves. One could imagine this to be again unspecific heterogeneous primary nucleation, paired with an adsorption of more the aggregation prone Aβ42 monomers onto Aβ40 seeds, effectively reducing the free monomer concentration and hence slowing aggregation. Most importantly, however, these effects are small compared to the effects observed in the aggregation from a mixture of monomers of both proteins and hence do not represent the dominant and therefore most important interactions of Aβ40 and Aβ42.
